# A Power-Law Dependence of Bacterial Invasion on Mammalian Host Receptors

**DOI:** 10.1371/journal.pcbi.1004203

**Published:** 2015-04-16

**Authors:** Tae J. Lee, Jeffrey Wong, Sena Bae, Anna Jisu Lee, Allison Lopatkin, Fan Yuan, Lingchong You

**Affiliations:** 1 Department of Biomedical Engineering, Duke University, Durham, North Carolina, United States of America; 2 Center for Genomic and Computational Biology, Duke University, Durham, North Carolina, United States of America; 3 Center for Systems Biology, Duke University, Durham, North Carolina, United States of America; University of Freiburg, GERMANY

## Abstract

Pathogenic bacteria such as *Listeria* and *Yersinia* gain initial entry by binding to host target cells and stimulating their internalization. Bacterial uptake entails successive, increasingly strong associations between receptors on the surface of bacteria and hosts. Even with genetically identical cells grown in the same environment, there are vast differences in the number of bacteria entering any given cell. To gain insight into this variability, we examined uptake dynamics of *Escherichia coli* engineered to express the invasin surface receptor from *Yersinia*, which enables uptake via mammalian host β_1_-integrins. Surprisingly, we found that the uptake probability of a single bacterium follows a simple power-law dependence on the concentration of integrins. Furthermore, the value of a power-law parameter depends on the particular host-bacterium pair but not on bacterial concentration. This power-law captures the complex, variable processes underlying bacterial invasion while also enabling differentiation of cell lines.

## Author Summary

Uptake of bacteria by mammalian cells is highly variable within a population of host cells and between host cell types. A detailed but unwieldy mechanistic model describing individual host-pathogen receptor binding events is captured by a simple power-law dependence on the concentration of the host receptors. The power-law parameters capture characteristics of the host-bacterium pair interaction and can differentiate host cell lines. This study has important implications for understanding the accuracy and precision of therapeutics employing receptor-mediated transport of materials to mammalian hosts.

## Introduction

Pathogenic bacteria such as *Yersinia pseudotuberculosis* and *Listeria monocytogenes* exploit a latent phagocytic activity in gut epithelia to pass into deeper tissues that are optimal for survival and proliferation [1]. Particularly well studied is the 'zipper' phagocytic process used by *Yersinia*: Envelopment of a bacterium by a host membrane is aided by successive binding events between bacterial surface receptors called invasins and cognate β_1_-integrins exposed on the surface of host intestinal multifold cells (M-cells) in the gut lumen [2,3].

Zipper-mediated phagocytosis can be broken down into three successive steps: The first step is contact and adherence; second is phagocytic cup formation; and the final stage is phagocytic cup closure. Contact does not require active alterations to host actin cytoskeleton but rather hinges on strong associations between bacterial receptors and host ligands. In contrast, the creation and resolution of the phagocytic cup is a dynamic process involving numerous signal transduction and structural genes coordinated in receptor clustering, membrane phospholipid redistribution, and cytoskeletal re-organization [4] (S1 Table). A particularly critical aspect is the high affinity of invasin for β_1_-integrins, which promotes phagocytosis by increasing cell-surface adhesion [5] and outcompetes extracellular matrix proteins for enough host integrins [6] to sustain bacterial engulfment.

A striking observation emerging from these studies is the cell-cell variability in bacterial uptake: Isogenic host cells cultivated under the same conditions show differences in the number of bacteria that will invade. Past studies have reported that this variability manifests as bimodal uptake dynamics, where a fraction of mammalian cells take up bacteria while the other fraction is devoid of bacteria [7–10]. This property is not limited to cultured experiments since *Yersinia* introduced into the intestines of mice are often found in islands of M-cells surrounded by bacteria-free regions and that the number of bacteria in infected cells spans a wide range [11]. Such differences may arise from the stochastic nature of cellular interactions [12,13]. Also, the uptake may have resulted from pre-existing, long-lived differences in host cell properties (e.g. cell surface, signal transduction network, cytoskeleton) that have stochastic [14] and deterministic [15] origins.

The complexity in bacterial uptake presents a challenge for explaining variability. Here, to characterize the fundamental property of bacterial uptake, we employ kinetic modeling and experiments that distill a simple power-law, relating uptake probability—the amount of bacteria per host cell scaled by the bacteria concentration—with host receptor levels. Our study demonstrates that a simplified model of successive binding events that occur in the zipper mechanism is sufficient to generate an ultrasensitive, threshold-dependent response to host receptors. Thus, minute cell-cell differences in host receptors are amplified into large differences in uptake. We describe how different hosts and bacterial strains translate into different power-law parameters which serves as the basis of a novel, *operational* definition of cell type.

## Results

### Variability in invasin-mediated bacterial uptake

We modified a previous cell culture protocol to measure bacterial uptake by HeLa human cervical cancer-derived cells [16]. In particular, we engineered non-pathogenic *E*. *coli* to express invasin from *Yersinia pseudotuberculosis* [17] (Materials and Methods). Invasin-mediated bacterial entry via binding with b1-integrins is one of the best-studied zipper-mechanism systems and has been used in a number of biological and potential clinical applications [7,9,18,19]. Remarkably, binding of invasins to mammalian host receptors is sufficient to facilitate entry into non-phagocytic mammalian cells. In fact, non-pathogenic bacteria expressing invasins [20] or even beads coated with invasins [21] can enter into mammalian host cells. Thus, the entry dynamics of this engineered bacterial system can be attributed to the interactions between invasin and host receptors. To track and quantify uptake in individual hosts, we engineered *E*. *coli* to constitutively express a green fluorescent protein (GFP). In each experiment, the engineered bacteria were co-cultured with HeLa cells for 90 minutes in well-mixed conditions to mitigate the effects of heterogeneity in the bacterial population. In addition, the mixture was co-incubated in the presence of sub-lethal gentamicin before washing and measurement (S1A–S1C Fig). This gentamicin treatment served two purposes by inhibiting bacterial growth: First, it reduced differences between bacterial numbers and our calculation of MOI that could arise during co-culture. Second, it minimized alterations in GFP signals due to bacterial growth.

**Fig 1:**
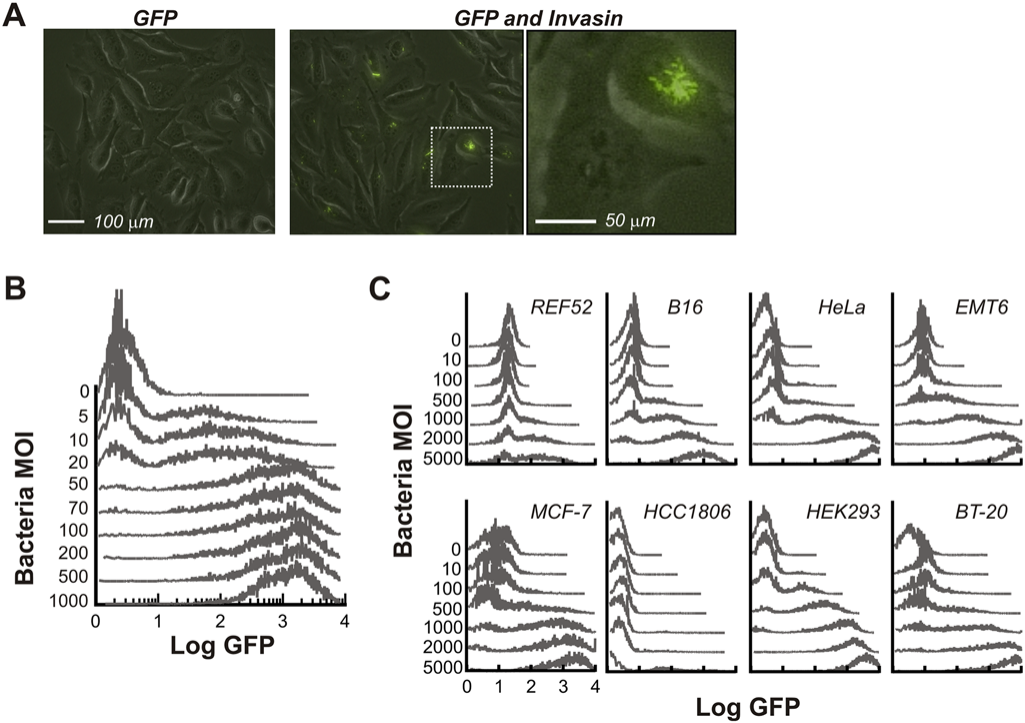
Cell-cell variability in invasin-mediated bacterial uptake. (**A**) Microscope image of phase and GFP signals from HeLa human cervical cancer cells co-cultured with *E*. *coli* harboring plasmids directing expression of (*left*) GFP only or (*center*, right) GFP and invasin (*pSCT7Inv*) at a multiplicity of infection (MOI) of 100 per mammalian cell for 60 minutes in the presence of gentamicin. Image on right is a magnification of boxed region. (**B and C**) Flow cytometry signals from *E*. *coli* (GFP/Invasin) incubated with (B) HeLa cells or (C) the indicated cell line as described in (A).

Consistent with observations reported in the literature [18,22], fluorescence microscopy confirmed drastic cell-cell variability in bacterial uptake by HeLa cells (Fig 1A). Some cells were devoid of bacteria, whereas the others each contained a wide range. This property was consistent with flow cytometry measurements (Fig 1B): At intermediate bacterial concentrations, a bimodal distribution of GFP fluorescence arises where within a single population there exist both uninfected cells (i.e. low mode) and infected cells (i.e. high mode). Even within the infected subpopulation, there was drastic variability in fluorescence, indicating a wide range of bacterial numbers in individual cells. We note that the characteristics of bacterial uptake depended on the bacterial multiplicity of infection (MOI) as well as the host cell type (Fig 1C). Uptake in all the cell lines we tested, most of which are cancer models, was positively correlated with MOI but the amount of uptake was quite variable.

Bacterial uptake variability within an infected population of cells has been speculated to arise from cell-cell variability in host surface membrane properties [11]. A trivial explanation is that variability in uptake reflects a wide, bimodal distribution of host β_1_-integrins or GFP signals in bacteria. However, immunolabeling experiments revealed a relatively narrow, unimodal distribution of β_1_-integrins across HeLa cells (S1D Fig). Similarly, GFP signals in bacteria showed a tight and unimodal distribution (S2 Fig). Hence, we hypothesized that the dynamics arising from zipper-like interactions may be responsible for generating the observed bimodal uptake from a unimodal β_1_-integrin distribution.

### A model of zipper-mediated bacterial uptake

**Fig 2:**
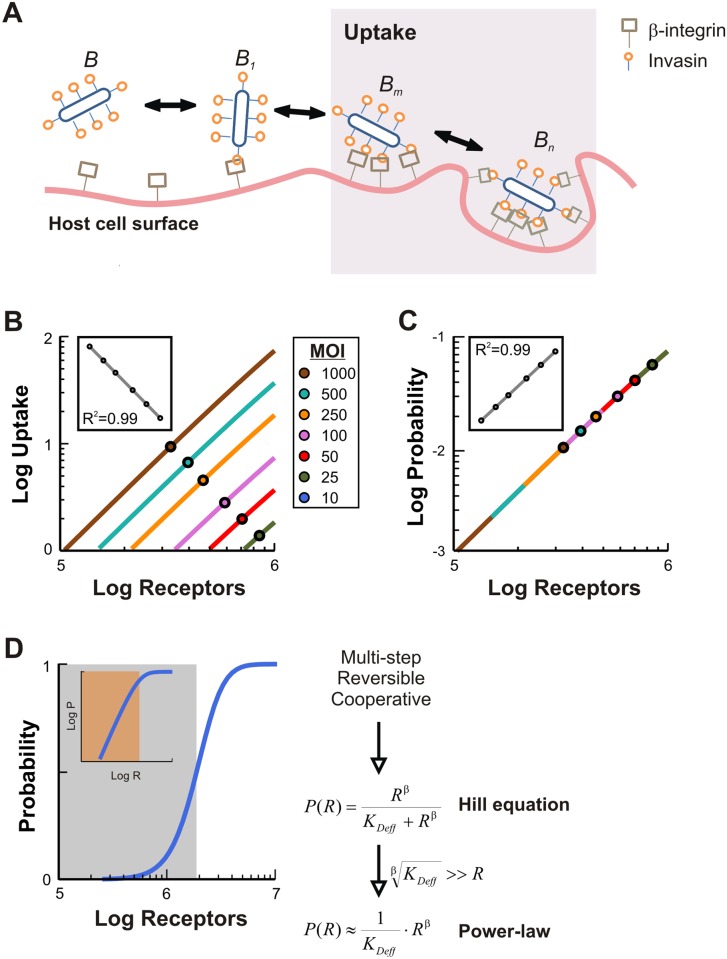
Zipper-mediated uptake can be described by a power-law. (**A**) Schematic of a 3-stage zipper model. Free bacteria (B) reversibly bind to β_1_-integrins in three stages: Unstable, singly bound (B_1_); stable attachment via a minimal number of interactions (B_m_); and a maximal number of interactions (B_n_). Uptake is defined as the sum of B_m_ and B_n_. (**B**) Simulated bacterial uptake per cell as a function of total β_1_-integrins ranging from 10^5^ to 10^6^ per cell for various MOI. Lines indicate the linear dependence of bacterial uptake on receptor concentration and circles indicate mean bacterial uptake in the infected host cells for each bacterial MOI. Inset shows linear fit to means. (**C**) The dependence of the uptake probability by a single bacterium on the host receptor concentration is independent of the bacterial concentration. Note that the uptake curves for larger MOI span a larger region of the same trajectory. The uptake probability is defined as the overall bacterial uptake normalized with respect to the total bacterial MOI. Inset shows power-law fit to means. (**D**) Power-law dependence arises in a limiting case of a more general Hill-type dose response. The zipper mechanism is a cooperative, multistep, and reversible process well-described by a Hill function. A power-law is a limiting case when the receptor concentration is less than the scaled effective dissociation constant i.e. R<<*K*_*Deff*_^1/β^ (blue shaded region). This condition is met when a highly cooperative, multistep, reversible process reduces the likelihood of finding bacteria in intermediate states. This regime corresponds to the linear region in a log-log plot (shaded region in inset).

To examine the mechanistic basis of the variable bacterial uptake, we developed a kinetic model consisting of a set of ordinary differential equations that capture two important aspects of the zipper mechanism (Fig 2A, supporting text (S1 Text) and tables (S1–S4) Tables). First, invasin-integrin interactions are inherently cooperative [23] as sequential binding events are increasingly likely due to host-pathogen proximity and the stabilizing role of invasin-integrin clustering [21]. Second, bacteria must maintain a minimum number of invasin-integrin interactions to remain stably attached to host cells.

We make the simplifying assumption that bacteria and β_1_-integrins interact as if they were in a homogeneous, well-mixed system. Furthermore, rather than explicitly describing every single receptor binding event, we divide the uptake process into three reversible stages (i.e. the 3-stage model). Initially, bacteria bind weakly to a small number of β_1_-integrins (state B_1_) and these initial interactions increase the avidity of subsequent binding events. In the second stage, weakly-bound bacteria can adhere in a more stable fashion by interacting with an intermediate number of β_1_-integrins (state B_m_), which we assume is sufficient for successful adherence even after repeated washing. In the final stage, a maximal number of β_1_-integrin interactions can be formed (state B_n_), representing the saturation of invasin receptors and a state that corresponds to bacterial engulfment and internalization [2,3]. Thus, we define bacterial uptake in this study is as the sum of bacteria achieving at least the intermediate binding state (i.e. sum of B_m_ and B_n_) to represent both stably-adhered and internalized bacteria. In fact, immunostaining of a single infected host cell with anti-LPS shows both stably-adhered and fully internalized bacteria (S3 Fig). We note that the number of β_1_-interactions depend on the amount of invasins expressed in bacteria cells, with successful adherence and internalization likely requiring a large number of interactions. Thus, rather than modeling each binding event explicitly to match experimental data, our goals with the 3-stage and the full model are to explore the conceptual basis for the experimentally observed variability in bacterial uptake. Indeed, the qualitative behaviors of our full model, in which each binding step is explicitly modeled, are not especially dependent upon the nominal values of intermediate (m = 10) or maximal number (n = 100) of interactions.

### A power-law summarizes uptake dependence on host receptors

Consistent with experimental measurements, our model predicts that both the mean uptake per cell and the fraction of the population infected increase with the bacterial MOI (Figs 2B and S4A). Simulation results show that these behaviors arise from a threshold relationship between uptake and host receptor number. In particular, host cells with fewer than threshold numbers of receptors do not take up bacteria; above a threshold, uptake is positively correlated with the number of host receptors. Since we assume a bi-molecular binding reaction between bacteria and mammalian receptors for the initial binding step, a higher bacterial concentration would require a lower mammalian receptor concentration to enable bacterial uptake. Thus, bacterial concentration modulates overall uptake while reducing the minimal threshold of host receptors. For example, uptake only occurs in cells with more than 6∙10^5^ β_1_-integrins at 25 MOI (Fig 2B; dark green) whereas only 1∙10^5^ β_1_-integrins are needed at 1000 MOI (Fig 2B; brown).

The threshold response produced by the zipper model indicates a sensitive dependence of bacterial uptake on bacterial concentration. For a given host receptor concentration, our model predicts that the bacterial uptake increases linearly (in log-log scale) with the bacterial concentration (Fig 2B, inset). This property could be expected as we have assumed independent binding of bacteria to receptors. In other words, from the perspective of a single bacterium, its uptake would solely depend on the host receptor concentration.

Consistent with this notion, when scaled with respect to the corresponding MOI values, the different dose responses in Fig 2B collapse into a single curve, which is approximately linear (Fig 2C). This curve summarizes how uptake depends on host receptor number. In essence, increasing bacterial MOI extends the uptake curve into a lower range of host receptor concentrations, as evidenced by a downward shift in the mean. Importantly, the sensitive threshold relationship and unified uptake trajectory were recapitulated by a zipper model explicitly describing all 100 binding/unbinding steps, indicating these behaviors are not specific to the simpler 3-stage model (S4B Fig).

The approximately linear relationship between the logarithm of uptake and host receptors indicates the power-law dependence. Indeed, the inset in Fig 2D show a fit by the equation logP = β·logR—logK_Deff_, where β and *K*_*Deff*_ are fitted parameters. Sequential perturbation of zipper model parameters shows that these changes can be mapped to changes in both power-law parameters (S4C Fig). In particular, changes to the dissociation constants for binding of B and B_1_ to receptors had the greatest effect. Altogether this analysis shows that a simple power-law provides a concise summary of zipper-mediated uptake.

How does such a simple dependence arise? Highly cooperative binding processes can often be approximated by the Hill equation [24], in which the fraction of particle binding as a function of the log of the input follows a characteristic sigmoidal trajectory. Zipper model simulations reveal that uptake probability as a function of receptor behaves in this characteristic fashion according to P = R^β^ / (K_Deff_ + R^β^) (Fig 2D). A power-law arises if the receptor concentration is much lower than the effective dissociation constant (i.e. K_Deff_ >> R^β^). A large *K*_*Deff*_ can arise from the reversible, multi-step nature of uptake that makes achieving the uptake state less likely than if it were a single binding event.

### The probability of invasin-mediated uptake is invariant

To test the predicted power-law, we needed to measure, in single host cells, the concentration of β_1_-integrins in addition to the bacterial uptake (as measured by GFP). To this end, we used a non-activating antibody to fluorescently label β_1_-integrins [25,26]. In principle, labeling with antibody may inhibit bacterial uptake by serving as competitive inhibitor. To examine impact of antibody on uptake, we also extended the model to account for sequestration of β_1_-integrins (S5A Fig). Modeling (S5B Fig) and experiments (S5C and S5D Fig) reveal that indeed, antibody addition could result in reduced uptake (over 40% reduction at > = 1.5 μg/mL). We chose a relatively low concentration of antibody (0.15 μg/mL) that reports the relative amount of receptors on host cells while not having a severe effect on bacterial uptake (S5E Fig). While antibody-bound receptors may not be directly involved in bacterial uptake, we reasoned that, at a sufficiently low concentration, the antibody-bound receptors may be used as a proxy reporter for the relative amount of β_1_-integrins.

**Fig 3:**
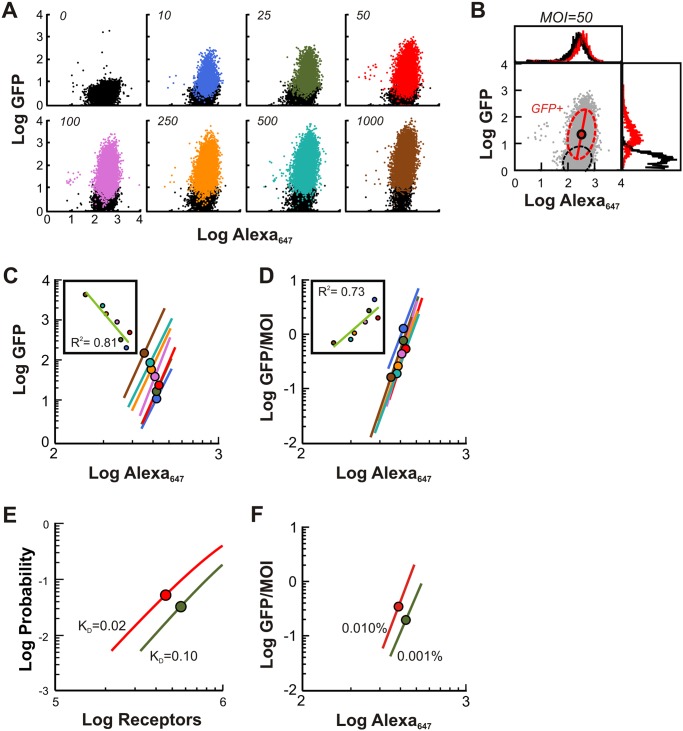
Uptake of bacteria is independent of one another. (**A**) Flow cytometry measurements of bacterial uptake at various MOI. HeLa cells were labeled with β_1_-integrin antibody for 60 minutes, washed, co-incubated with GFP-expressing *E*. *coli* grown in 0.1% arabinose to induce high-level of invasin expression from the *pBACr-AraInv* plasmid, along with fluorescent-conjugated secondary antibody (Alexa_647_) for 90 minutes prior to washing. Scatter plots show the relationship between uptake (Log GFP) and β_1_-integrins (Log Alexa_647_) in individual cells. Each uptake distribution for MOI>0 is bimodal with infected cells (GFP+ mode) coexisting with those devoid of bacteria (GFP- mode). The two modes are designated by a Gaussian mixture model (see (B)), each mode parameterized by a mean and a standard deviation. Colored and black points indicate GFP+ and GFP- subpopulations, respectively. (**B**) Processing of data for MOI = 50 in (A). The expectation-maximization (EM) algorithm was used to probabilistically assign cells into either GFP- sub-population (black) or GFP+ sub-population (red). Histograms summarize data from each respective channel; Principle components represented by ellipse and major axis superimposed on scatter plot (circle). The half-length of the major axis (i.e. from the center to a vertex) represents twice the standard deviation in that direction. (**C**) Correlation between uptake and host receptor levels. Using flow cytometry data in (A), each line approximates the dependence of the bacterial uptake in the GFP+ subpopulation on the host receptor concentration for the corresponding MOI. The length of each line is 4 times the standard deviation along the major axis. Mean indicated by circle. Inset shows linear fit on means. (**D**) Uptake probability. Using flow cytometry data in (A), GFP values were scaled by their respective MOI and then processed as in (C). These curves approximate the dependence of the uptake probability per bacterium on the host receptor concentrations. (**E**) Simulations showing the dependence of uptake on the host receptor number per cell at bacterial MOI of 250 for two values of the invasin dissociation constant (K_D_ = k_rB1_/k_fB1_). (**F**) Experimental modulation of invasin expression. HeLa cells were incubated with *E*. *coli* (GFP/Invasin) grown in the indicated concentrations of arabinose to vary invasin expression, which leads to enhanced binding of bacteria to the host receptors. Shown are the major axes and mean of GFP+ subpopulations scaled by their respective MOI.

After co-culture of increasing amounts of GFP-expressing *E*. *coli* harboring a plasmid permitting arabinose-inducible control of *invasin* (*pBACr-AraInv*) with HeLa cells, flow cytometry was conducted to obtain data shown in Fig 3A. In the absence of induction, bacteria carrying *pBACr-AraInv* cannot infect host cells, which was reported previously [27] and observed by us by co-incubating HeLa cells with uninduced bacteria (S6A–S6C Fig). We further showed that bacterial uptake increased with increasing concentrations of invasin induction (S6D–S6G Fig). Further validating the construct, our qPCR experiments showed significantly increased invasin expression in arabinose-induced bacteria compared to uninduced bacteria (S7 Fig). Consistent with previous modeling (Fig 2B), collected data suggested an increase in the fraction and overall amount of uptake with bacterial MOI.

To simplify the data for comparison, a centroid and mean level of β_1_-integrins at a given fraction of infected host cells were computationally estimated from each flow cytometry sample at different MOIs (Fig 3B). In each population, single-cell measurements revealed a positive correlation between the infected host cells and the corresponding level of β_1_-integrins (Fig 3C). With increasing bacterial MOI, the major axes of GFP+ subpopulations were shifted towards higher GFP levels, consistent with an overall increase in uptake, and towards lower β_1_-integrin levels, indicative of a reduced threshold of receptors required for uptake. When GFP values were scaled with their respective MOI for the fraction of infected host cells, the curves collapsed into a single linear line that has high correlation (Fig 3D), similar to our previous simulation results (Fig 2C). This striking observation indicates that the correlation between the uptake and host receptor levels follows a power law as predicted by model.

These behaviors were reproduced using the GFP-expressing *E*. *coli* strain with *invasin* expressed from a different promoter (*pSCT7Inv*; S5F–S5H Fig). A final prediction of our model is that the location of probability curves could be modulated by varying the invasin *K*_*Deff*_ (Fig 3E). Indeed, when the bacterial MOI was fixed at 200, increasing arabinose shifted the scaled GFP values to higher GFP and lower host receptor levels (Fig 3F).

### Characteristic power-law parameters describe uptake in different cell lines

**Fig 4:**
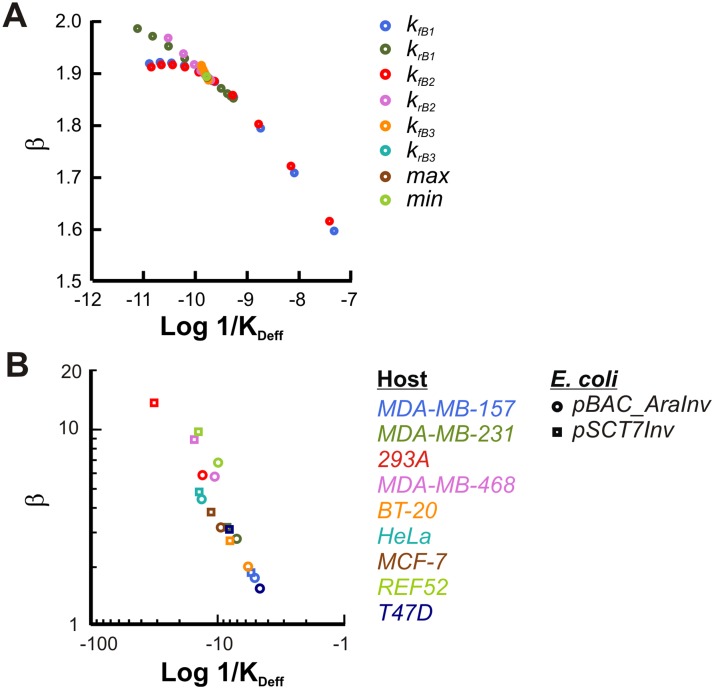
Host and pathogen specific power-law parameters. (**A**) Mapping of 3-stage model parameters on power-law parameters. Each set of markers shows the effect of increasing a zipper model parameter over two orders of magnitude centered on a base value. Zipper model simulations were performed at MOI (500) and a power-law of the form P = R^β^/K_Deff_ was fit to scaled output. Simulated data presented in S4C Fig (**B**) Power-law parameters for different hosts and bacterial strains. Mammalian cells were co-cultured with the indicated strain of GFP-expressing E. coli at 1000 MOI as described in (A). Raw data presented in S8D Fig

Our analysis demonstrates that, for a wide range of system parameters, zipper-mediated uptake is captured by a power law. Changes in many kinetic parameters associated with the zipper mechanism can be mapped to changes in the power-law parameters (Fig 4A; simulated data in S4C Fig). In a typical cell, changes in zipper-mechanism parameters reflect the changes in either the bacteria or the host cells. For instance, the initial binding rate (k_fB1_) between bacteria and host cells is affected by factors including expression levels of invasin and β_1_-integrins along with the prevalence of β chain integrins in host cells. For the same bacterial strain, different host cell types would likely lead to different kinetic parameters for the zipper mechanisms, and thus different corresponding power-law parameters. If this notion is correct, the power-law parameters can be used as a *quantitative phenotype* for a host cell type in a specific growth environment.

To test this hypothesis, we measured the uptake dynamics of our engineered bacteria (*E*. *coli* expressing arabinose-inducible invasin) in several mammalian cell lines, grown under the same condition. In theory, we expect that different uptake probability dose responses would be measured at different bacterial MOI to share a given power-law trajectory. However, measurements done at low MOI values would likely be prone to fluctuations due to the stochastic nature of the interaction between small numbers of bacteria and mammalian cells. This would then lead to greater uncertainty in the estimated power-law parameters.

In fact, our experimental measurements show this property. In particular, we treated several cell lines with increasing MOI. And these measurements showed that the power-law parameter values stabilized with increasing MOI, which likely resulted from more accurate measurement and analysis when more cells were in the GFP+ mode (S8A–S8C Fig). As such, for more extensive analysis of different cell lines, we chose a high MOI for bacterial infection.

Results in Fig 4B reveal that power-law parameters β and 1/K_Deff_ for different cell lines infected with bacteria at 1000 MOI appeared to fall along a single trajectory. This is reminiscent of modeling analysis showing that perturbation to zipper model parameters modulates power-law parameter values along the same, restricted path (Fig 4A). In addition, for each host cell line, β and *K*_*Deff*_ were lower for *E*. *coli* harboring *pBAC-AraInv* relative to *pSCT7Inv* plasmid, possibly resulting from the lower invasin expression from *pBAC-AraInv* under these conditions.

The approximately linear correlation between **β** and 1/K_Deff_ can be understood by examining the equation governing the emergent power-law (Fig 2D). Every set of perturbations results in quasi-parallel lines for a range of specific receptor concentrations, thus enabling the data collapse over several orders of magnitude for biologically relevant values (Fig 4A). However, as receptor concentration approaches zero, the linear fit breaks down and all lines approach a quasi-constant locus (S9A Fig). Therefore, when examining the correlation between **β** and 1/K_Deff_, we can construct an inverse linear regression fit (S9B Fig) such that the slope and intercept of this line correspond to the locus of convergence for receptors and for probability uptake. We are therefore left with a nontrivial dependent relationship between the power-law fitted parameters, which fall along the linear correlation between **β** and 1/K_Deff,_:$\;\beta \;=\;\frac{\log\left(P^{*}\right)}{\log\left(R^{*}\right)}-\frac{\log\left(\frac{1}{K_{Deff}}\right)}{\log\left(R*\right)}$.

## Discussion

Zipper-mediated phagocytosis entails coordination of a diverse set of host cellular processes. Bacterial uptake through this mechanism has been found to be highly variable, even within isogenic cells grown under tightly controlled experimental conditions [18,22,28]. In general, heterogeneous behaviors have been believed to arise from the stochastic nature of biochemical reactions [29] and differences in the local microenvironment for each cell, for example, the placement of neighboring cells [30]. Phagocytosis is particularly complex as it is affected by quantitative changes in host signal transduction molecules [31], cytoskeletal dynamics [32], particle receptor affinity [6], host receptor density, particle size, and shape [33]. It has been suggested that the hundreds of signaling molecules are potentially involved [33]. The sheer complexity presents a seemingly daunting challenge for attempts to incorporate detailed molecular knowledge into a theoretical model with predictive power [34].

We show that, despite the complexity, bacterial uptake by a 'zipper' mechanism can be modeled as sequential binding between bacterial invasin ligands to host receptors. Our detailed, quantitative, single-cell measurements revealed that, in the same population of host cells, the fraction of host cells infected with bacteria increases in a monotonic fashion with the levels of host β_1_-integrins—irrespective of MOI. Likewise, our modeling results demonstrate a positive correlation between invasin-mediated uptake of *E*. *coli* and the relative abundance of available β_1_-integrins expressed by hosts.

A counterintuitive, striking notion from our analysis is that uptake may be distilled into a simple empirical relation defined by two lumped parameters. Indeed, phenomenological approaches have been routinely used when detailed description is impractical, for example, oxygen binding [24], gene regulation [35], and bacterial growth [36]. Similarly, power-law relations have a rich history in the description of scale-invariant processes in nature [37]. Here we show that an ultrasensitive host-pathogen relationship explains a large proportion of the variation in pathogen invasion. Our results demonstrate generation of broad cell-cell variability in bacterial uptake by a Hill-like mechanism involving surface receptor interactions, though other mechanisms can contribute to this variability. For example, there could be a positive feedback in host cell cytoskeletal signaling that determine phagocytosis of bound bacteria.

The emergence of a power-law correlation suggests that microscopic understanding does not necessarily enhance our ability to describe some lumped system behavior [38]. Though simple, the power-law description of uptake is robust in describing invasin-mediated bacterial uptake by mammalian cells, and the extracted power-law parameters can be useful in distinguishing pairs of *E*. *coli* strains (expressing varying levels of invasion) and mammalian cell lines. This represents a complementary, simple and efficient means to characterize cell physiology. Each perturbation to the system produces a unique set of power-law parameters; a library of these parameters can be collected for easily accessible sample analysis. Also, this notion can be extended to other pathogens and particles that utilize a multi-step, reversible binding process such as viruses. Similar to bacterial uptake by host cells, viruses also employ receptor-mediated endocytosis to use biological machinery in the host cells for them to replicate [39]. This behavior plays an important role in viral infections including Influenza and Rubella [40]. By providing simple predictions for multi-step receptor-mediated uptake, the power-law description can enable us to capture cellular dynamics without identifying interconnected processes involved in each step.

## Methods

### Plasmid construction

GFP expression plasmid pTetGFP was created by fusing a PCR-derived fragment of the *gfp* gene into *pPROTet*.*E* using the KpnI and BamHI enzyme digestion sites. To construct the plasmid pSCT7Inv, *the invasin* gene was PCR amplified from pRI203 [17] with BamHI and EcoRI enzyme digestion sites at the end and inserted into similar sites of a plasmid that contained a Kanamycin resistant maker gene and sc101 replication origin. The arabinose-inducible plasmid, pBACr-AraInv, is provided by Dr. J Christopher Anderson, UC Berkeley [27].

### Bacterial growth

Top10F’ bacterial strain was transformed with either pTetGFP only or with either pSCT7Inv or pBACr-AraInv. Cells were grown overnight at 37°C in Luria-Bertani (LB) and diluted in Dulbecco’s modified eagle medium (D-MEM; GIBCO® Cat. No. 31053–036) supplemented with 2% L-glutaminine (Cat. No. 25030–164), 1% sodium pyruvate (Cat. No. 11360–070), and 0.02% bovine growth serum (BGS) until their absorbance reading (Abs_600_) on a plate reader was approximately 1.0. At this absorbance, the number of bacteria in 1μL of the culture was approximately 2.0 x 10^6^.

### Mammalian cell culture and infection

All mammalian cells (provided by Dr. Joseph Nevins, Duke University) were grown in D-MEM supplemented with 2% L-glutaminine, 1% sodium pyruvate, and 10% BGS. Prior to co-culture with bacteria, cells were seeded in 6-well plates at approximately 2.0 x 10^5^/well, and were allowed to grow overnight in D-MEM with 10% BGS. Typically, bacterial overnight cultures were co-incubated with mammalian host cells in the presence of gentamicin (50μg/ml) for 90 minutes to allow infection. Then the co-cultured cells were rigorously washed twice with phosphate buffered saline (PBS) to remove bacteria in suspension, trypsinized to detach mammalian host cells for flow cytometry, fixed with PBS supplemented with 1% formaldehyde, 0.33% BGS and 0.001% of sodium pyruvate and assayed for their GFP signals by flow cytometry (FACSCanto II, Becton Dickinson). For fluorescent microscopy (DMI6000 microscope, Leica), cells were imaged immediately after rigorous washing with PBS twice. For quantification of β_1_-integrin levels, mammalian cells were pre-incubated with a monoclonal antibody (Cat No. MAB2253, Millipore) followed by detection with anti-mouse antibody conjugated to Alexa_647_ (Cat No. A21237, Invitrogen).

### RNA extraction and qPCR

A single colony of *E*. *coli* Top10F’ strain containing pBACr-AraINV and pTetGFP was cultured overnight at 37°C in LB media for 16 hours. Sub-culturing were performed with 100-fold dilution in LB with appropriate antibiotics (Kan and Cm) and induced with 0.1% arabinose when appropriate. Induced and uninduced samples were cultured for additional 5 hours at 37°C, then RNA samples were isolated from the cell cultures using RNeasy Mini Kit (QIAGEN, cat. no. 74104) in combination with RNAprotect Bacteria Reagent (QIAGEN, cat. no. 76506). Using Power SYBR Green RNA-to-C_T_ 1-Step Kit (Life Technologies, cat. no.4389986), qPCR was conducted with primers targeting invasin (forward primer: CTCACTCAATGGTGGGCGAT; reverse primer: CATACCAAGGAGCCAGCCAA) and FFH was used as a reference gene for normalization (forward primer: TGTGACGAATAGAGAGCGCC; reverse primer: GGCCAATACGGCAAAAGCAT). The standard curve method for relative quantification was used.

### Immunostaining of surface-bound bacteria

Approximately 2×10^4^ HeLa cells/well were seeded onto glass coverslips placed in a 24 well plate. On the following day, HeLa cells were then infected with invasin-expressing *E*. *coli* at an MOI of 200 by plate rotation at an rpm of 50, in a 37^°^C, 5% CO2 humidified incubator for 90min. After infection, HeLa cell were washed twice with phosphoate buffered saline (PBS) and fixed with 3% formaldehyde/0.025% glutaraldehyde at room temperature for 10 min. HeLa cells were then blocked with 5% BSA in PBS for 30 min at room temperature and stained with anti-LPS *E*. *coli* (Abcam). Secondary antibody conjugated to Alexa Fluor 555 (Life technologies), and Hoechst 33342 (Life Technologies) where then incubated for 30min.

### Mathematical model

Our kinetic models (3-stage or 100-stage) each consist of a set of coupled ordinary differential equations (ODEs). The parameters are obtained either from the literature or fitted from experimental data. Numerical simulations and data analysis were performed using Matlab (Natick, MA). The detailed models are described in the supporting text (S1 Text) and tables (S1–S4 Tables).

## Supporting Information

S1 Fig Calibration of bacterial uptake assay. (**A**) Effect of gentamicin on *E*. *coli* growth. Bacterial growth in 50 μg/mL gentamicin measured through turbidity (OD_600_). *E*. *coli* (GFP/Invasin) expressing GFP and expressing Invasin from the plasmid pSCT7Inv were grown in a plate reader at 37°C in 1ml Dulbecco’s minimal media supplemented with 10% bovine growth serum (BGS) with the indicated number of bacteria. (**B**) Flow cytometry of bacterial GFP associated with mammalian cells. HeLa cells were co-incubated with *E*. *coli* (GFP/Invasin) at a multiplicity of infection (MOI) of 2000 in the absence or presence of 50 μg/mL gentamicin for the indicated amount of time prior to measurement. (**C**) Statistical summary of data from (B). The expectation-maximization (EM) algorithm was applied to GFP signals in order to assign cells to either a GFP+ sub-population (colored) or a GFP- sub-population. (**D**) Flow cytometry detection of surface β_1_-integrins. HeLa cells incubated with either control antibody (actin) or antibody against β_1_-integrin along with fluorescent secondary antibody (Alexa_647_).
(TIF)

S2 Fig Expression of induced GFP. Flow cytometry data for GFP expression. pBacr-AraInv and pTetGFP plasmids were introduced into Top10F' E. *coli*, enabling the control of Invasin expression through addition of arabinose and control of GFP expression through ATC, respectively. Top10F’ cells containing only pBacr-AraInv were used for control. Cultures were grown overnight at 37°C in LB for 16 hours and sub-cultures were performed with 100-fold dilution from overnight culture. (A) The control strain was grown without arabinose induction for comparison, and Top10F’ containing pBacr-AraInv and pTetGFP was either (B) uninduced, or (C) induced with. 1% arabinose and 100ng/ml ATC to induce Invasin and GFP. The mean expression level of GFP in the induced sample (C) is approximately three magnitudes higher than that measured in the uninduced sample (B). Also, the basal level expression in the cells containing only pBacr-AraInv (A) is also at least two magnitudes smaller than that in the induced sample (C). All three panels in the figure show tight and unimodal distributions of GFP expression.
(TIF)

S3 Fig Immunostaining to identify bacteria on host surface and internalized bacteria. HeLa cells, infected with *E*. *coli* expressing invasin and GFP at an MOI of 200 for 90 minutes, were washed with PBS twice, fixed with paraformaldehyde, and immunostained with an anti-LPS antibody to identify bacterial cell wall (red color) and Hoechst to identify DNA (blue color). In the merged image, yellow dots (indicated by white arrows) represent GFP-expressing bacteria that were immunostained in red, suggesting that they are surface-bound. Green dots represent fully internalized bacteria that were prevented from immunostaining by the mammalian cell wall. The presence of both green and yellow bacteria suggests that invasin-expressing bacteria can adhere and invade into mammalian host cells.
(TIF)

S4 Fig Zipper model simulations. (**A**) Simulations using a 3-stage zipper model with a lognormal input distribution (top; n = 2000) of receptor numbers at various MOI (bottom). (**B**) Simulations using a 100-stage zipper model. (*Left*) A 100-stage zipper model that explicitly describes each bacterial binding state produces qualitatively the same results as does the 3-stage model (compare with Fig 2B). Horizontal axis indicates total number of host receptors per cell. Uptake is defined as the total amount of bacteria bound to at least 10 or more host receptors. Mean indicated by circles. (*Right*) Similar to the prediction by the 3-stage model, the 100-stage model also predicts that an approximate power-law captures the dependence of the uptake probability by a single bacterium on the concentration of the host receptors. The uptake probabilities are calculated by scaling data on left with the respective MOI. (**C**) Mapping of kinetic parameters associated with the 3-stage model to power-law parameters. Zipper model simulations were performed and the uptake was calculated for MOI (500). A power-law of the form P = R^β^/K_Deff_ was fit to data for different values of each zipper model parameter. The zipper model parameters are normalized with respect to their base values (S4 Table).
(TIF)

S5 Fig Antibody-mediated detection of host surface β_1_-integrins. (**A**) Relationship between bacterial binding and amount of surface β_1_-integrins immunolabeling. Abbreviations: A-R—antibody-receptor complex. (**B**) Simulations. Relationship between uptake and A-R on host cells in the presence of the indicated amount of antibody for β_1_-integrins. (**C**) Flow cytometry measurements of bacterial uptake in HeLa cells in the presence of increasing concentration of the antibody. HeLa cells were pre-incubated for 60 minutes with the indicated amount of β_1_-integrin antibody and subsequently co-cultured with GFP-expressing *E*. *coli* also Invasin from the plasmid pSCT7Inv at 500 MOI and secondary antibody (Alexa_647_) for an additional 90 minutes prior to washing and measurement. The EM algorithm was used to assign cells into the GFP+ mode (colored). (**D**) Statistical summary of flow cytometry data in (C). Statistics from the EM algorithm were used to extract the mean (circle) and major axes (lines) of the GFP+ mode. Numbers beside the means indicate inhibition calculated as the mean GFP signal relative to the mean GFP signal at the lowest antibody concentration (GFP_o_ = 1.5e-6 μg/mL) (i.e. [GFP—GFP_o_]/GFP_o_). (**E**) Simulations. Cell receptors are pre-incubated with 0.15 μg/mL of antibody for 60 minutes of simulation time followed by 90 minutes with the indicated MOI of bacteria. (*Left*) Relationship between bacterial uptake and A-R. (*Right*) Relationship between probability (uptake/MOI) and A-R calculated from simulated data. These results show that the dependence of bacterial uptake on the concentration of antibody-bound receptors is qualitatively the same as the dependence on total receptor (Fig 2B and 2C). (**F**) Flow cytometry demonstrating that qualitative relationship between uptake and host receptors is recapitulated with a different bacterial strain. Experiments were performed as described in S5C Fig in the presence of 0.15 μg/mL of β_1_-integrin antibody and the indicated MOI of GFP-expressing *E*. *coli* also expressing INV from the plasmid pSCT7Inv. EM algorithm was used to assign cells to the GFP+ subpopulation (colored). (**G**) Fractional uptake. For each MOI sample in (F), data points in GFP+ mode within 2.5 standard deviations of the mean Alexa_647_ signal were extracted, placed into 15 contiguous bins, and the fraction of GFP+ cells were plotted. (**H**) Uptake and probability. Shown for each MOI sample in (F) are the *(left)* major axes and mean or (*right*) GFP values scaled by bacterial MOI.
(TIF)

S6 Fig Invasin induction by arabinose. HeLa cells (A) were co-cultured with bacteria harboring pTetGFP alone (B), pTetGFP and pBACr-AraInv without arabinose induction (C), or with induction at the indicated concentrations of arabinose (D-G). The blue line denotes auto-fluorescent GFP signal from HeLa cells, and is overlaid for comparison with GFP signals in other conditions (shown in red). S6A–S6C Fig show similar GFP distributions, suggesting that bacteria without invasin expression cannot invade HeLa cell and that arabinose induction is required for bacterial uptake. S6D and S6E Fig show the dependence of bacterial uptake on arabinose induction.
(TIF)

S7 Fig Expression of induced invasin. Quantification of *invasin* expression induced by arabinose. Top10F’ cells containing pBACr-AraINV and pTetGFP were either uninduced or induced with 0.1% arabinose and harvested after 5 hours. qPCR was conducted with primers targeting invasin and FFH was used as a reference gene for normalization. The fold change in the average expression level of invasin was then calculated using the standard curve method for relative quantification. The average expression level of invasin in the induced sample was 25 times greater than that in the uninduced sample. The average level of invasin expression is calculated from either 3 or 4 replicates.
(TIF)

S8 Fig Uptake in different host cells types using different bacterial strains. (**A**) Flow cytometry. Mammalian cells were labeled with β_1_-integrin antibody for 60 minutes, washed, then co-incubated with GFP-expressing *E*. *coli* also expressing Invasin from pBAC-AraInv (cultured in 0.1% arabinose) at the indicated MOI along with fluorescent-conjugated secondary antibody (Alexa_647_) for 90 minutes prior to washing. Scatter plots show the relationship between uptake (Log GFP) and β_1_-integrins (Log Alexa_647_) in individual cells. (**B**) Statistical summary of data in (A). Shown are the major axes and mean of GFP+ subpopulations from (A). (**C**) Power-law parameters for calculated from samples presented in (A) and data in (B). (**D**) Flow cytometry. Indicated mammalian cells were co-cultured with the indicated GFP-expressing strain of *E*. *coli* (pSCT7Inv, pBACr-AraInv) as described in (A). Shown on right are the major axes of GFP+ subpopulations scaled by their respective MOI.
(TIF)

S9 FigLinear correlation of power-law parameters.(**A**) For each parameter, the zipper model simulates several perturbations over two orders of magnitude. This results in quasi-parallel lines consistent with the emergence of the power-law, analogous to that of Fig 2B; the spread of the distribution of each set of lines is determined by the model’s sensitivity to each respective parameter. Lines extended from the power-law simulation results no longer remain parallel away from biologically relevant receptor concentrations. As the power-law breaks down, each line approximately approaches a common point of convergence (R*, P*). The subset shown above consists of one line chosen from each perturbation. (**B**) Apparent linear correlation between fitted power-law parameters is defined by the quasi-constant locus (R*, P*), which determines the slope and intercept for the lines of best fit. Every point will fall along the same trajectory, as described by equation $\;\beta \;=\;\frac{\log\left(P^{*}\right)}{\log\left(R^{*}\right)}-\frac{\log\left(\frac{1}{K_{Deff}}\right)}{\log\left(R*\right)}$.(TIF)

S1 Text Description of mathematical models. (DOCX)

S1 Table Mechanisms implicated in invasin-mediated uptake. (DOCX)

S2 Table Reaction terms. (DOCX)

S3 Table Ordinary differential equations. (DOCX)

S4 Table Parameters. (DOCX)

## References

[1] Cossart P, Sansonetti PJ (2004) Bacterial invasion: the paradigms of enteroinvasive pathogens. Science 304: 242–248. 15073367 10.1126/science.1090124

[2] Isberg RR, Barnes P (2001) Subversion of integrins by enteropathogenic Yersinia. J Cell Sci 114: 21–28. 11112686 10.1242/jcs.114.1.21

[3] Isberg RR, Hamburger Z, Dersch P (2000) Signaling and invasin-promoted uptake via integrin receptors. Microbes Infect 2: 793–801. 10955960 10.1016/s1286-4579(00)90364-2

[4] Swanson JA (2008) Shaping cups into phagosomes and macropinosomes. Nat Rev Mol Cell Biol 9: 639–649. 10.1038/nrm2447 18612320 PMC2851551

[5] Van Nhieu GT, Isberg RR (1991) The Yersinia pseudotuberculosis invasin protein and human fibronectin bind to mutually exclusive sites on the alpha 5 beta 1 integrin receptor. J Biol Chem 266: 24367–24375. 1837020

[6] Tran Van Nhieu G, Isberg RR (1993) Bacterial internalization mediated by beta 1 chain integrins is determined by ligand affinity and receptor density. EMBO J 12: 1887–1895. 8491181 10.1002/j.1460-2075.1993.tb05837.x PMC413409

[7] Laner A, Goussard S, Ramalho AS, Schwarz T, Amaral MD, et al (2005) Bacterial transfer of large functional genomic DNA into human cells. Gene Therapy 12: 1559–1572. 15973438 10.1038/sj.gt.3302576

[8] Garcia-Del Portillo F (2008) Heterogeneity in tissue culture infection models: a source of novel host-pathogen interactions? Microbes and Infection 10: 1063–1066. 10.1016/j.micinf.2008.07.004 18662799

[9] Critchley-Thorne RJ, Stagg AJ, Vassaux G (2006) Recombinant Escherichia coli expressing invasin targets the Peyer's patches: The basis for a bacterial formulation for oral vaccination. Molecular Therapy 14: 183–191. 16581299 10.1016/j.ymthe.2006.01.011

[10] Abraham SN, Beachey EH, Simpson WA (1983) Adherence of Streptococcus-Pyogenes, Escherichia-Coli, and Pseudomonas-Aeruginosa to Fibronectin-Coated and Uncoated Epithelial-Cells. Infection and Immunity 41: 1261–1268. 6411621 10.1128/iai.41.3.1261-1268.1983 PMC264634

[11] Clark MA, Hirst BH, Jepson MA (1998) M-cell surface beta1 integrin expression and invasin-mediated targeting of Yersinia pseudotuberculosis to mouse Peyer's patch M cells. Infect Immun 66: 1237–1243. 9488419 10.1128/iai.66.3.1237-1243.1998 PMC108039

[12] Balazsi G, van Oudenaarden A, Collins JJ (2011) Cellular decision making and biological noise: from microbes to mammals. Cell 144: 910–925. 10.1016/j.cell.2011.01.030 21414483 PMC3068611

[13] Wu M, Su R-Q, Li X, Ellis T, Lai Y-C, et al (2013) Engineering of regulated stochastic cell fate determination. Proceedings of the National Academy of Sciences. 10.1073/pnas.1305423110 PMC3696818 23754391

[14] Sigal A, Milo R, Cohen A, Geva-Zatorsky N, Klein Y, et al (2006) Variability and memory of protein levels in human cells. Nature 444: 643–646. 17122776 10.1038/nature05316

[15] Snijder B, Pelkmans L (2011) Origins of regulated cell-to-cell variability. Nat Rev Mol Cell Biol 12: 119–125. 10.1038/nrm3044 21224886

[16] Miliotis MD (1991) Acridine orange stain for determining intracellular enteropathogens in HeLa cells. J Clin Microbiol 29: 830–831. 1716265 10.1128/jcm.29.4.830-831.1991 PMC269882

[17] Isberg RR, Voorhis DL, Falkow S (1987) Identification of invasin: a protein that allows enteric bacteria to penetrate cultured mammalian cells. Cell 50: 769–778. 3304658 10.1016/0092-8674(87)90335-7

[18] Grillot-Courvalin C, Goussard S, Huetz F, Ojcius DM, Courvalin P (1998) Functional gene transfer from intracellular bacteria to mammalian cells. Nat Biotechnol 16: 862–866. 9743121 10.1038/nbt0998-862

[19] Xiang S, Fruehauf J, Li CJ (2006) Short hairpin RNA-expressing bacteria elicit RNA interference in mammals. Nat Biotechnol 24: 697–702. 16699500 10.1038/nbt1211

[20] Isberg RR, Falkow S (1985) A single genetic locus encoded by Yersinia pseudotuberculosis permits invasion of cultured animal cells by Escherichia coli K-12. Nature 317: 262–264. 2995819 10.1038/317262a0

[21] Dersch P, Isberg RR (1999) A region of the Yersinia pseudotuberculosis invasin protein enhances integrin-mediated uptake into mammalian cells and promotes self-association. EMBO J 18: 1199–1213. 10064587 10.1093/emboj/18.5.1199 PMC1171211

[22] Garcia-Del Portillo F (2008) Heterogeneity in tissue culture infection models: a source of novel host-pathogen interactions? Microbes Infect 10: 1063–1066. 10.1016/j.micinf.2008.07.004 18662799

[23] Ivaska J, Heino J (2000) Adhesion receptors and cell invasion: mechanisms of integrin-guided degradation of extracellular matrix. Cell Mol Life Sci 57: 16–24. 10949578 10.1007/s000180050496 PMC11146885

[24] Weiss JN (1997) The Hill equation revisited: uses and misuses. FASEB J 11: 835–841. 9285481

[25] Gonzalez AM, Gonzales M, Herron GS, Nagavarapu U, Hopkinson SB, et al (2002) Complex interactions between the laminin alpha 4 subunit and integrins regulate endothelial cell behavior in vitro and angiogenesis in vivo. Proc Natl Acad Sci U S A 99: 16075–16080. 12454288 10.1073/pnas.252649399 PMC138567

[26] Humphries MJ (2004) Monoclonal antibodies as probes of integrin priming and activation. Biochem Soc Trans 32: 407–411. 15157148 10.1042/BST0320407

[27] Anderson JC, Clarke EJ, Arkin AP, Voigt CA (2006) Environmentally controlled invasion of cancer cells by engineered bacteria. J Mol Biol 355: 619–627. 16330045 10.1016/j.jmb.2005.10.076

[28] Laner A, Goussard S, Ramalho AS, Schwarz T, Amaral MD, et al (2005) Bacterial transfer of large functional genomic DNA into human cells. Gene Ther 12: 1559–1572. 15973438 10.1038/sj.gt.3302576

[29] Kaern M, Elston TC, Blake WJ, Collins JJ (2005) Stochasticity in gene expression: from theories to phenotypes. Nat Rev Genet 6: 451–464. 15883588 10.1038/nrg1615

[30] Snijder B, Sacher R, Ramo P, Damm EM, Liberali P, et al (2009) Population context determines cell-to-cell variability in endocytosis and virus infection. Nature 461: 520–523. 10.1038/nature08282 19710653

[31] Alrutz MA, Isberg RR (1998) Involvement of focal adhesion kinase in invasin-mediated uptake. Proc Natl Acad Sci U S A 95: 13658–13663. 9811856 10.1073/pnas.95.23.13658 PMC24875

[32] Young VB, Falkow S, Schoolnik GK (1992) The invasin protein of Yersinia enterocolitica: internalization of invasin-bearing bacteria by eukaryotic cells is associated with reorganization of the cytoskeleton. J Cell Biol 116: 197–207. 1730744 10.1083/jcb.116.1.197 PMC2289272

[33] Tollis S, Dart AE, Tzircotis G, Endres RG (2010) The zipper mechanism in phagocytosis: energetic requirements and variability in phagocytic cup shape. BMC Syst Biol 4: 149 10.1186/1752-0509-4-149 21059234 PMC2991294

[34] Underhill DM, Ozinsky A (2002) Phagocytosis of microbes: complexity in action. Annu Rev Immunol 20: 825–852. 11861619 10.1146/annurev.immunol.20.103001.114744

[35] Bintu L, Buchler NE, Garcia HG, Gerland U, Hwa T, et al (2005) Transcriptional regulation by the numbers: models. Curr Opin Genet Dev 15: 116–124. 15797194 10.1016/j.gde.2005.02.007 PMC3482385

[36] Scott M, Gunderson CW, Mateescu EM, Zhang Z, Hwa T (2010) Interdependence of cell growth and gene expression: origins and consequences. Science 330: 1099–1102. 10.1126/science.1192588 21097934

[37] Gisiger T (2001) Scale invariance in biology: coincidence or footprint of a universal mechanism? Biol Rev Camb Philos Soc 76: 161–209. 11396846 10.1017/s1464793101005607

[38] Golan B, Brian M, Ilya N (2010) The simplicity of completion time distributions for common complex biochemical processes. Physical Biology 7: 016003 10.1088/1478-3975/7/1/016003 20026876

[39] Lakadamyali M, Rust MJ, Zhuang X (2004) Endocytosis of influenza viruses. Microbes and Infection 6: 929–936. 15310470 10.1016/j.micinf.2004.05.002 PMC2715838

[40] Pelkmans L, Helenius A (2003) Insider information: what viruses tell us about endocytosis. Current Opinion in Cell Biology 15: 414–422. 12892781 10.1016/s0955-0674(03)00081-4

