# Supplementary figures and images for: A Power-Law Dependence of Bacterial Invasion on Mammalian Host Receptors
Source: PLoS Comput Biol. 2015 Apr 16;11(4):e1004203. doi: 10.1371/journal.pcbi.1004203 (PMC4399907; doi:10.1371/journal.pcbi.1004203)

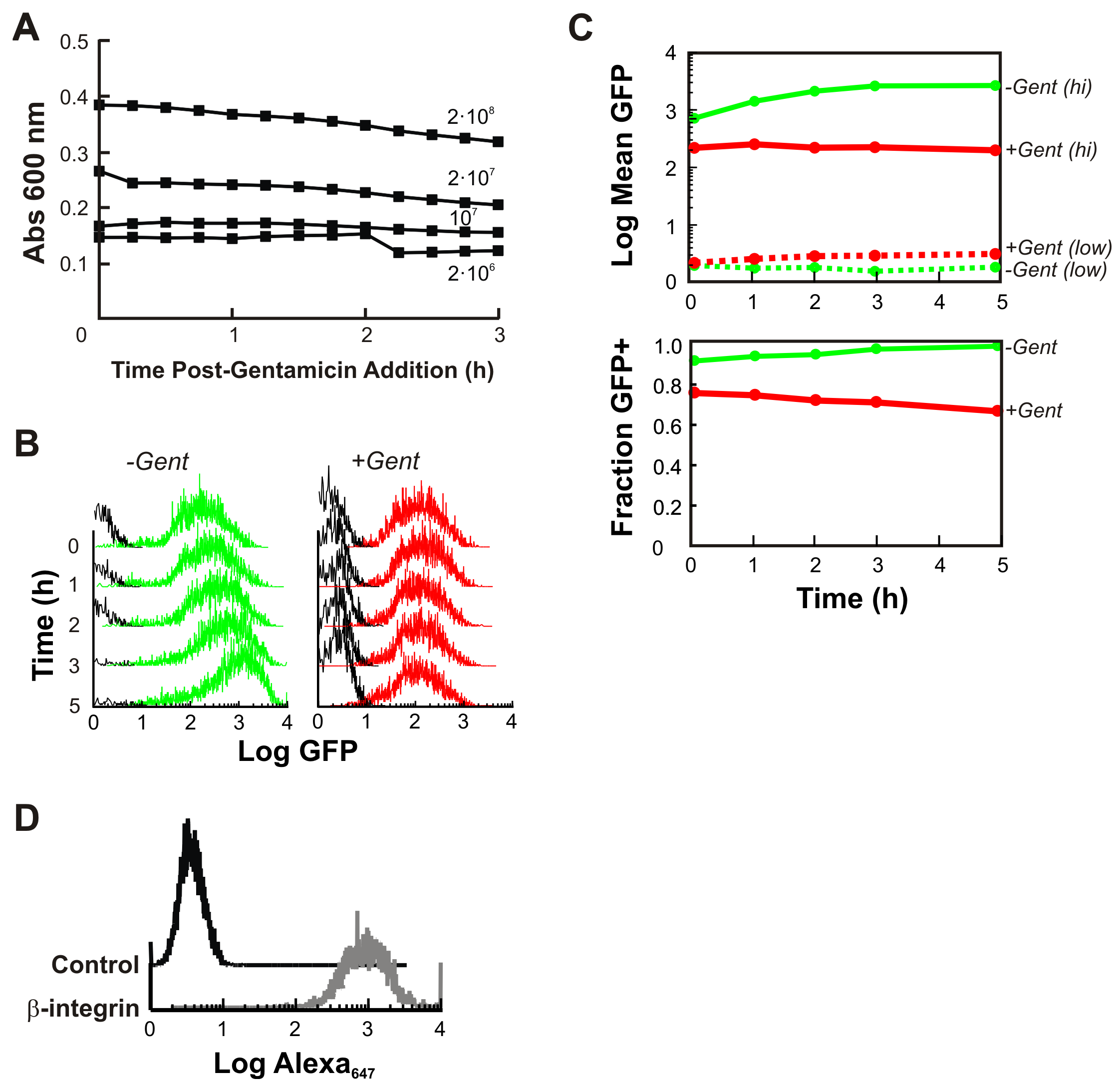

Supplement: S1 Fig — (A) Effect of gentamicin on E. coli growth. Bacterial growth in 50 μg/mL gentamicin measured through turbidity (OD600). E. coli (GFP/Invasin) expressing GFP and expressing Invasin from the plasmid pSCT7Inv were grown in a plate reader at 37°C in 1ml Dulbecco’s minimal media supplemented with 10% bovine growth serum (BGS) with the indicated number of bacteria. (B) Flow cytometry of bacterial GFP associated with mammalian cells. HeLa cells were co-incubated with E. coli (GFP/Invasin) at a multiplicity of infection (MOI) of 2000 in the absence or presence of 50 μg/mL gentamicin for the indicated amount of time prior to measurement. (C) Statistical summary of data from (B). The expectation-maximization (EM) algorithm was applied to GFP signals in order to assign cells to either a GFP+ sub-population (colored) or a GFP- sub-population. (D) Flow cytometry detection of surface β1-integrins. HeLa cells incubated with either control antibody (actin) or antibody against β1-integrin along with fluorescent secondary antibody (Alexa647). (TIF) [file pcbi.1004203.s001.tif]

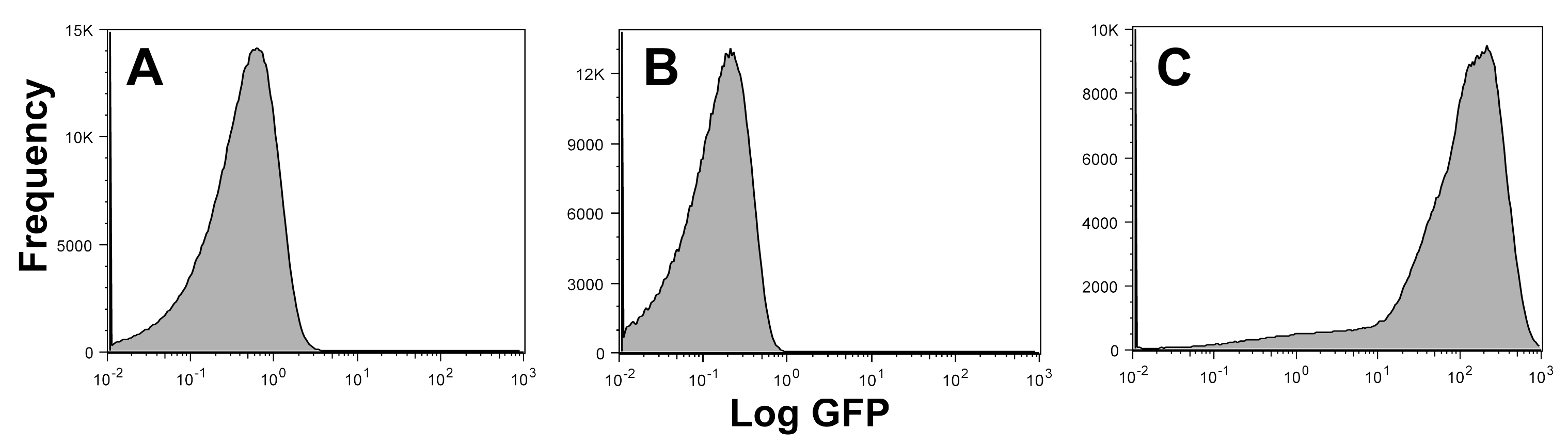

Supplement: S2 Fig — Flow cytometry data for GFP expression. pBacr-AraInv and pTetGFP plasmids were introduced into Top10F' E. coli, enabling the control of Invasin expression through addition of arabinose and control of GFP expression through ATC, respectively. Top10F’ cells containing only pBacr-AraInv were used for control. Cultures were grown overnight at 37°C in LB for 16 hours and sub-cultures were performed with 100-fold dilution from overnight culture. (A) The control strain was grown without arabinose induction for comparison, and Top10F’ containing pBacr-AraInv and pTetGFP was either (B) uninduced, or (C) induced with. 1% arabinose and 100ng/ml ATC to induce Invasin and GFP. The mean expression level of GFP in the induced sample (C) is approximately three magnitudes higher than that measured in the uninduced sample (B). Also, the basal level expression in the cells containing only pBacr-AraInv (A) is also at least two magnitudes smaller than that in the induced sample (C). All three panels in the figure show tight and unimodal distributions of GFP expression. (TIF) [file pcbi.1004203.s002.tif]

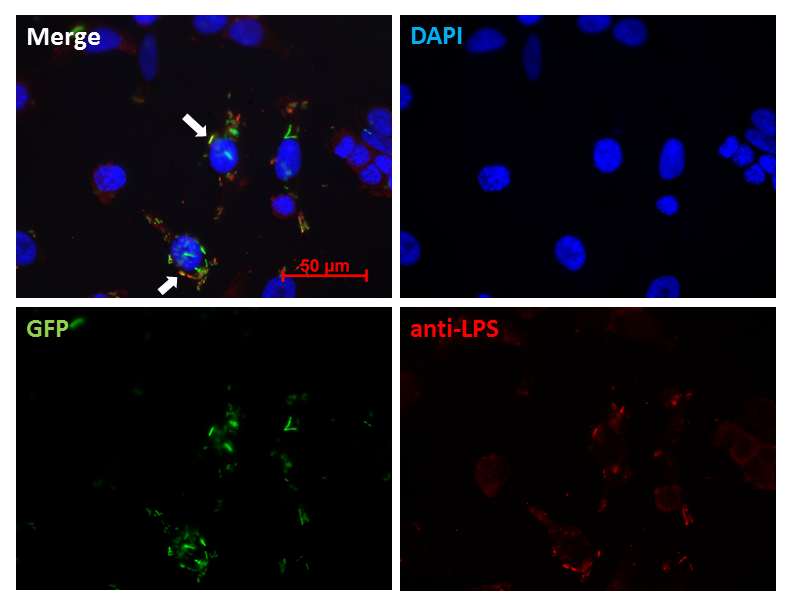

Supplement: S3 Fig — HeLa cells, infected with E. coli expressing invasin and GFP at an MOI of 200 for 90 minutes, were washed with PBS twice, fixed with paraformaldehyde, and immunostained with an anti-LPS antibody to identify bacterial cell wall (red color) and Hoechst to identify DNA (blue color). In the merged image, yellow dots (indicated by white arrows) represent GFP-expressing bacteria that were immunostained in red, suggesting that they are surface-bound. Green dots represent fully internalized bacteria that were prevented from immunostaining by the mammalian cell wall. The presence of both green and yellow bacteria suggests that invasin-expressing bacteria can adhere and invade into mammalian host cells. (TIF) [file pcbi.1004203.s003.tif]

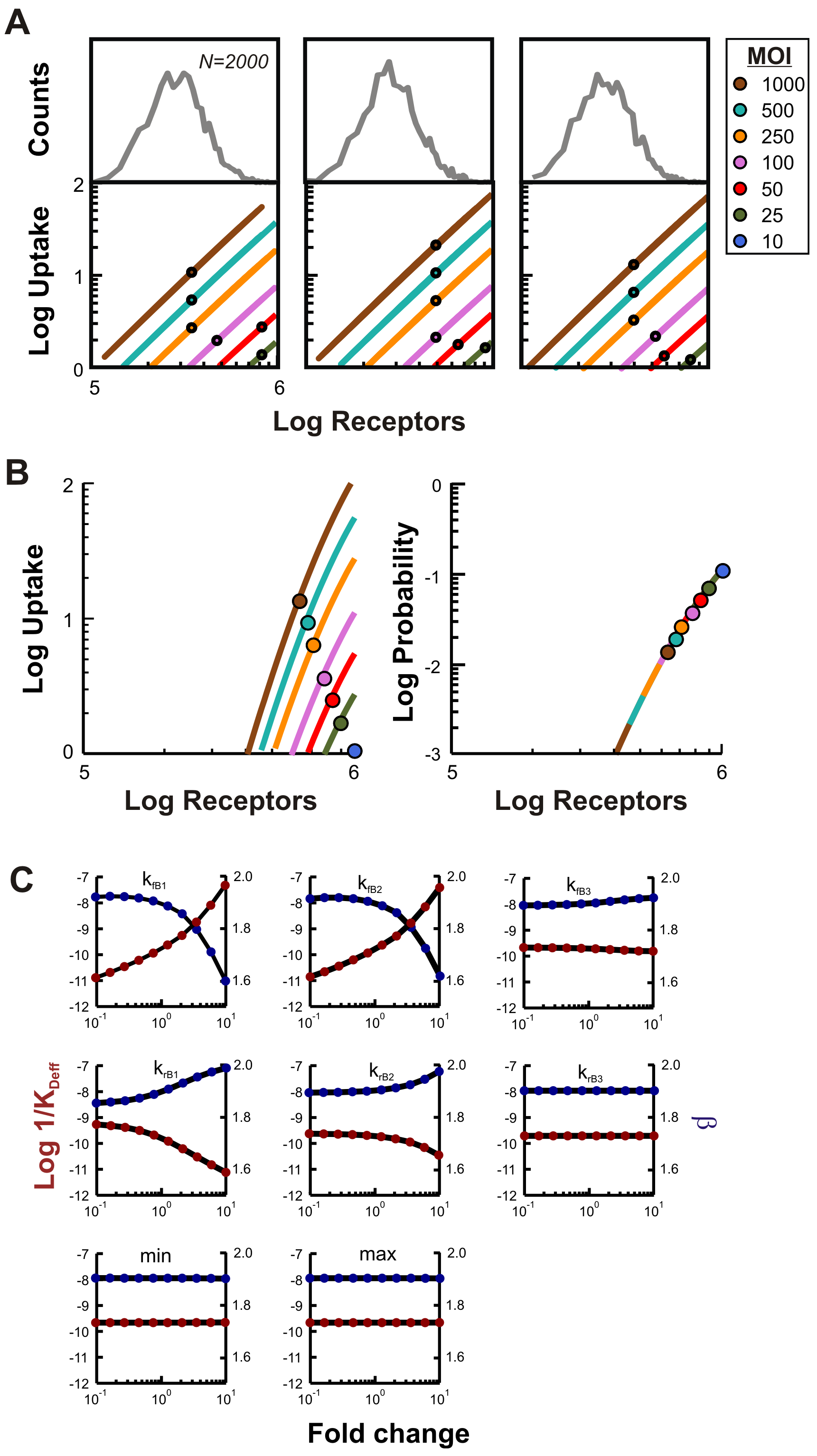

Supplement: S4 Fig — (A) Simulations using a 3-stage zipper model with a lognormal input distribution (top; n = 2000) of receptor numbers at various MOI (bottom). (B) Simulations using a 100-stage zipper model. (Left) A 100-stage zipper model that explicitly describes each bacterial binding state produces qualitatively the same results as does the 3-stage model (compare with Fig 2B). Horizontal axis indicates total number of host receptors per cell. Uptake is defined as the total amount of bacteria bound to at least 10 or more host receptors. Mean indicated by circles. (Right) Similar to the prediction by the 3-stage model, the 100-stage model also predicts that an approximate power-law captures the dependence of the uptake probability by a single bacterium on the concentration of the host receptors. The uptake probabilities are calculated by scaling data on left with the respective MOI. (C) Mapping of kinetic parameters associated with the 3-stage model to power-law parameters. Zipper model simulations were performed and the uptake was calculated for MOI (500). A power-law of the form P = Rβ/KDeff was fit to data for different values of each zipper model parameter. The zipper model parameters are normalized with respect to their base values (S4 Table). (TIF) [file pcbi.1004203.s004.tif]

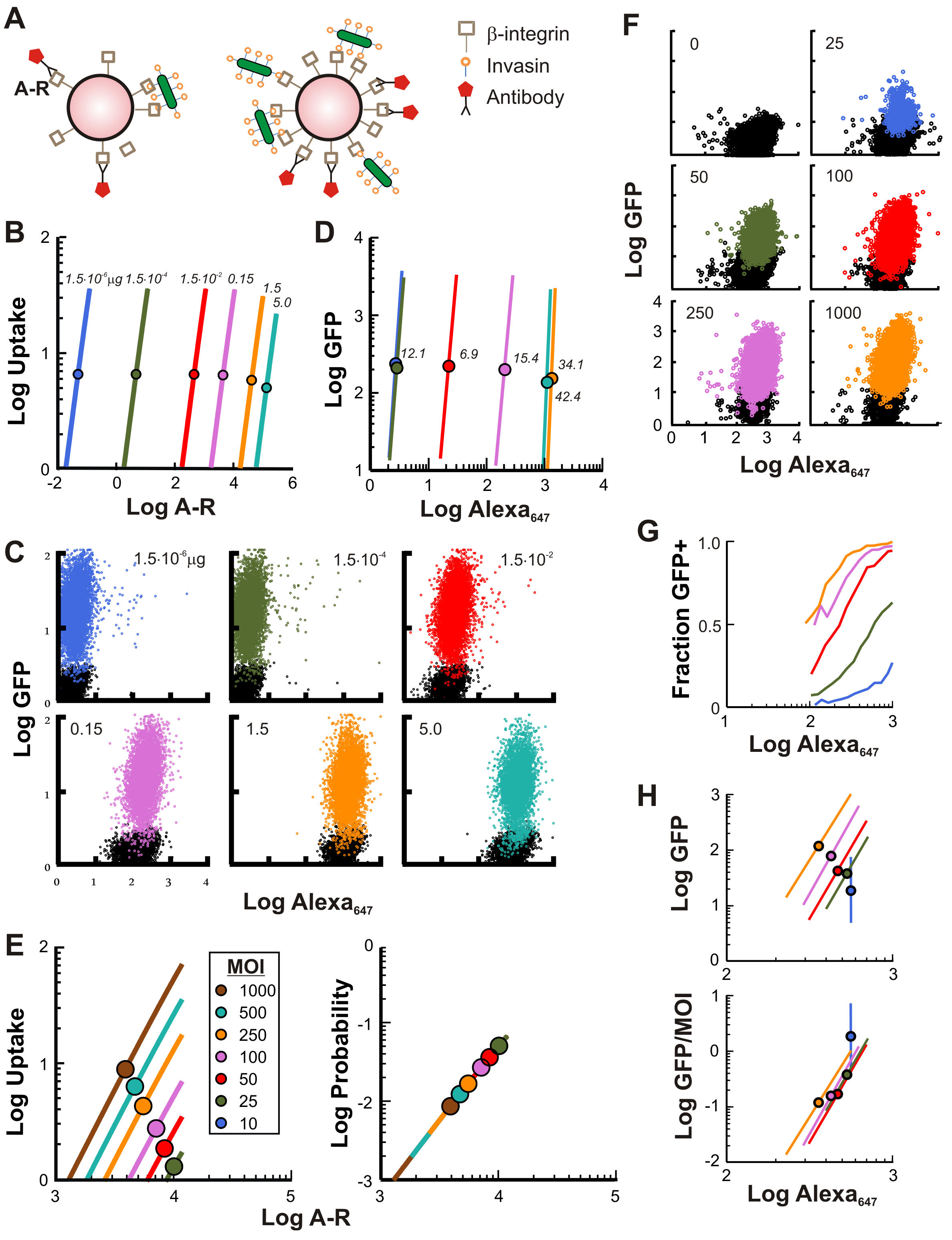

Supplement: S5 Fig — (A) Relationship between bacterial binding and amount of surface β1-integrins immunolabeling. Abbreviations: A-R—antibody-receptor complex. (B) Simulations. Relationship between uptake and A-R on host cells in the presence of the indicated amount of antibody for β1-integrins. (C) Flow cytometry measurements of bacterial uptake in HeLa cells in the presence of increasing concentration of the antibody. HeLa cells were pre-incubated for 60 minutes with the indicated amount of β1-integrin antibody and subsequently co-cultured with GFP-expressing E. coli also Invasin from the plasmid pSCT7Inv at 500 MOI and secondary antibody (Alexa647) for an additional 90 minutes prior to washing and measurement. The EM algorithm was used to assign cells into the GFP+ mode (colored). (D) Statistical summary of flow cytometry data in (C). Statistics from the EM algorithm were used to extract the mean (circle) and major axes (lines) of the GFP+ mode. Numbers beside the means indicate inhibition calculated as the mean GFP signal relative to the mean GFP signal at the lowest antibody concentration (GFPo = 1.5e-6 μg/mL) (i.e. [GFP—GFPo]/GFPo). (E) Simulations. Cell receptors are pre-incubated with 0.15 μg/mL of antibody for 60 minutes of simulation time followed by 90 minutes with the indicated MOI of bacteria. (Left) Relationship between bacterial uptake and A-R. (Right) Relationship between probability (uptake/MOI) and A-R calculated from simulated data. These results show that the dependence of bacterial uptake on the concentration of antibody-bound receptors is qualitatively the same as the dependence on total receptor (Fig 2B and 2C). (F) Flow cytometry demonstrating that qualitative relationship between uptake and host receptors is recapitulated with a different bacterial strain. Experiments were performed as described in S5C Fig in the presence of 0.15 μg/mL of β1-integrin antibody and the indicated MOI of GFP-expressing E. coli also expressing INV from the plasmid pSCT7Inv. EM algor [file pcbi.1004203.s005.tif]

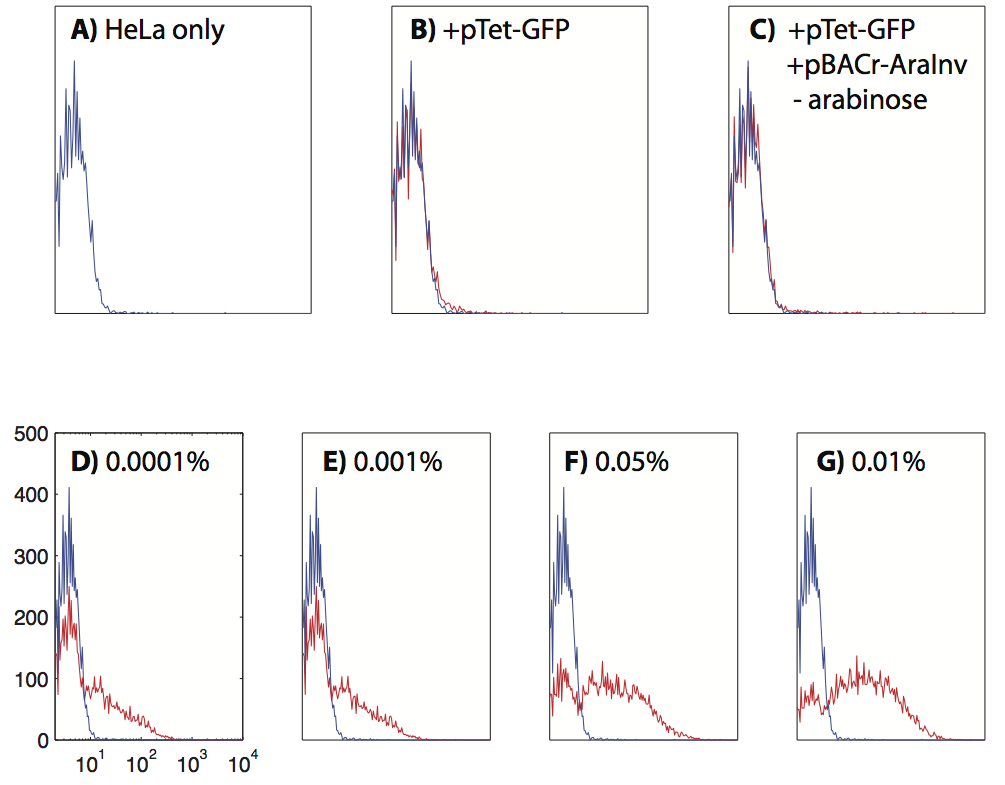

Supplement: S6 Fig — HeLa cells (A) were co-cultured with bacteria harboring pTetGFP alone (B), pTetGFP and pBACr-AraInv without arabinose induction (C), or with induction at the indicated concentrations of arabinose (D-G). The blue line denotes auto-fluorescent GFP signal from HeLa cells, and is overlaid for comparison with GFP signals in other conditions (shown in red). S6A–S6C Fig show similar GFP distributions, suggesting that bacteria without invasin expression cannot invade HeLa cell and that arabinose induction is required for bacterial uptake. S6D and S6E Fig show the dependence of bacterial uptake on arabinose induction. (TIF) [file pcbi.1004203.s006.tif]

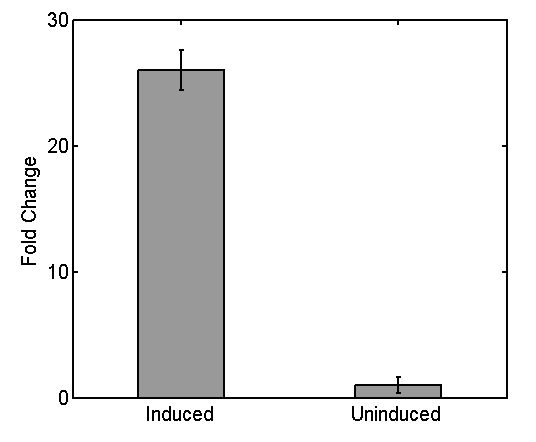

Supplement: S7 Fig — Quantification of invasin expression induced by arabinose. Top10F’ cells containing pBACr-AraINV and pTetGFP were either uninduced or induced with 0.1% arabinose and harvested after 5 hours. qPCR was conducted with primers targeting invasin and FFH was used as a reference gene for normalization. The fold change in the average expression level of invasin was then calculated using the standard curve method for relative quantification. The average expression level of invasin in the induced sample was 25 times greater than that in the uninduced sample. The average level of invasin expression is calculated from either 3 or 4 replicates. (TIF) [file pcbi.1004203.s007.tif]

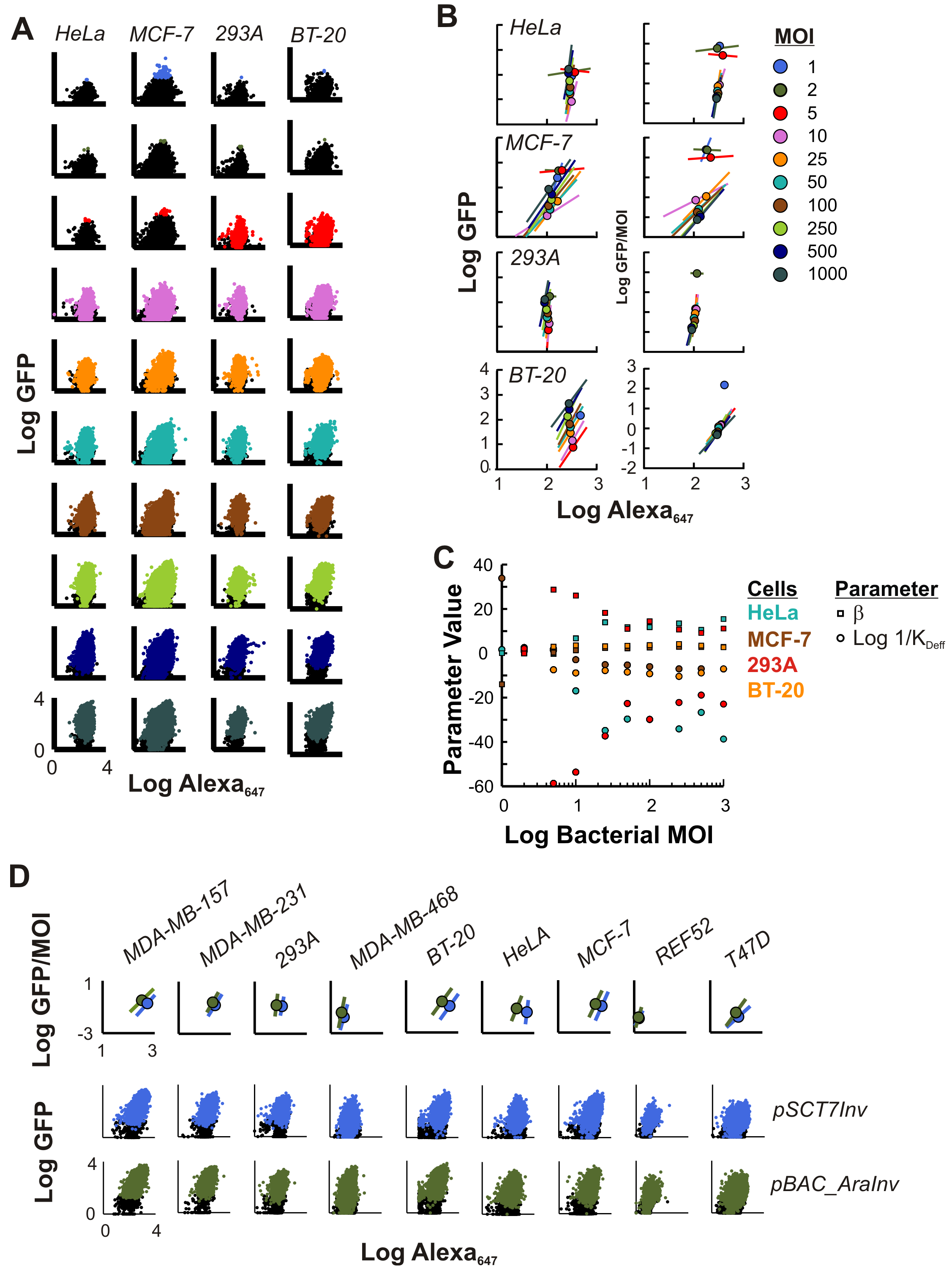

Supplement: S8 Fig — (A) Flow cytometry. Mammalian cells were labeled with β1-integrin antibody for 60 minutes, washed, then co-incubated with GFP-expressing E. coli also expressing Invasin from pBAC-AraInv (cultured in 0.1% arabinose) at the indicated MOI along with fluorescent-conjugated secondary antibody (Alexa647) for 90 minutes prior to washing. Scatter plots show the relationship between uptake (Log GFP) and β1-integrins (Log Alexa647) in individual cells. (B) Statistical summary of data in (A). Shown are the major axes and mean of GFP+ subpopulations from (A). (C) Power-law parameters for calculated from samples presented in (A) and data in (B). (D) Flow cytometry. Indicated mammalian cells were co-cultured with the indicated GFP-expressing strain of E. coli (pSCT7Inv, pBACr-AraInv) as described in (A). Shown on right are the major axes of GFP+ subpopulations scaled by their respective MOI. (TIF) [file pcbi.1004203.s008.tif]

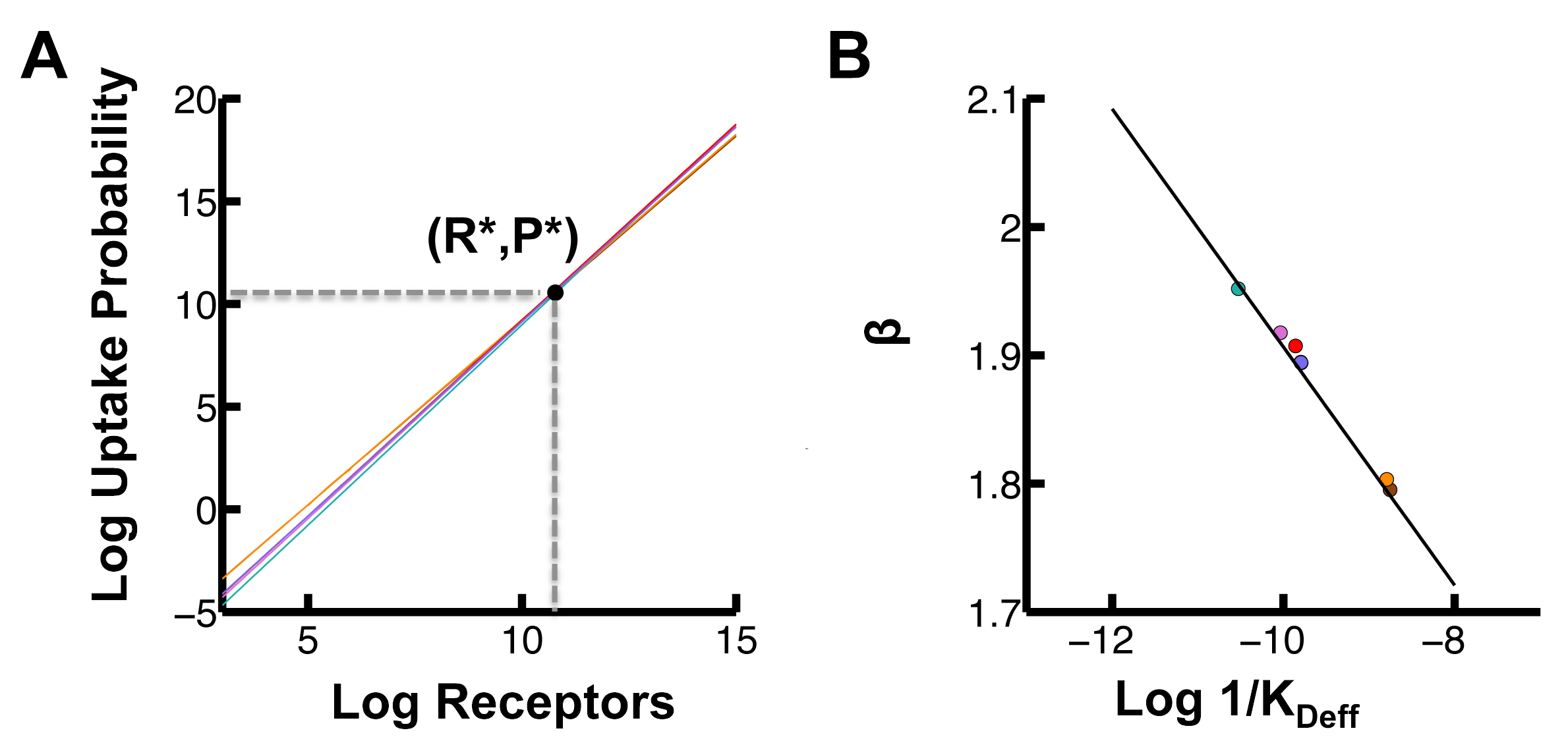

Supplement: S9 Fig — (A) For each parameter, the zipper model simulates several perturbations over two orders of magnitude. This results in quasi-parallel lines consistent with the emergence of the power-law, analogous to that of Fig 2B; the spread of the distribution of each set of lines is determined by the model’s sensitivity to each respective parameter. Lines extended from the power-law simulation results no longer remain parallel away from biologically relevant receptor concentrations. As the power-law breaks down, each line approximately approaches a common point of convergence (R*, P*). The subset shown above consists of one line chosen from each perturbation. (B) Apparent linear correlation between fitted power-law parameters is defined by the quasi-constant locus (R*, P*), which determines the slope and intercept for the lines of best fit. Every point will fall along the same trajectory, as described by equation β = logP*logR*-log1KDefflogR*. (TIF) [file pcbi.1004203.s009.tif]
