# Supplementary material for: A Power-Law Dependence of Bacterial Invasion on Mammalian Host Receptors
Source: PLoS Comput Biol. 2015 Apr 16;11(4):e1004203. doi: 10.1371/journal.pcbi.1004203 (PMC4399907; doi:10.1371/journal.pcbi.1004203)
Supplement: S1 Text — (DOCX) [file pcbi.1004203.s010.docx]

# Description of mathematical models

- Our kinetic model of the zipper mechanism involving mammalian β_1_-integrins and bacterial INV is modified from Perelson’s [1], which was used to describe multiple ligand-receptor interactions. We have developed a “full” model describing individual binding events as well as a “3-stage” model, which coarse grains host-receptor binding events into three stages (Table S2 and Table S3): (1) Bacteria bound to a single host receptor (B_1_), corresponding to weakly bound bacteria; (2) a minimum number of Invasin-integrin interactions (B_m_) allowing stable bacteria adherence sites, and (3) fully encapsulated bacteria (B_n_). Uptake is defined as the sum of bacteria achieving at least the minimally bound state (i.e. B_m_ and B_n_).
- The kinetic model assumes all reactants exist in a homogenous, well-mixed system. We base our estimates of host β_1_-integrin numbers upon the typical number of fibronectin receptors in fibroblasts (5·10^5^ per cell)[2]. Our cell culture protocol typically involves 2·10^5^ cells in a well-mixed reaction volume of 0.02 L. It can be shown that the average available number of β_1_-integrin receptors in a single well is on the order of 0.05 nM.
- As a further simplification, we do not model INV receptors expressed by bacteria explicitly but we assume that INV are not limiting. Thus, bacteria adopt different binding states (e.g. B_1_, B_m_, and B_n_) by associating with host β_1_-integrins sequentially. In the case of our cell culture conditions, the molar concentration of single *E. coli* is 8·10^-13^ nM and uptake occurs when the concentration of bacteria in at least the B_m_ state exceeds this value.
- For antibody calculations, we assumed a molecular weight of 88 kDa [3,4]. The concentration of 1μg/mL of antibody corresponds to ~6 nM.$\left[ \mathrm{Ab} \right]=\frac{Amount of Ab}{volume medium}*\frac{1}{Molecular weight}$ $\left[ \mathrm{Ab} \right]=\frac{\mathrm{AmountofAb}}{\mathrm{volumemedium}}*\frac{1}{\mathrm{Molecularweight}}$

**References**

1. Perelson AS. Receptor clustering on a cell surface. III. theory of receptor cross-linking by multivalent ligands: description by ligand states. Math Biosci. 1981;53: 1–39. doi:10.1016/0025-5564(81)90036-5

2. Akiyama SK, Yamada KM. The interaction of plasma fibronectin with fibroblastic cells in suspension. J Biol Chem. 1985;260: 4492–4500.

3. Gao J, Shattil SJ. An enzyme-linked immunosorbent assay to identify inhibitors of activation of platelet integrin αIIbβ3. J Immunol Methods. 1995;181: 55–64. doi:10.1016/0022-1759(94)00329-U

4. Wilkins JA, Li A, Ni H, Stupack DG, Shen C. Control of beta1 integrin function. Localization of stimulatory epitopes. J Biol Chem. 1996;271: 3046–3051.
