# Supplementary material for: A Power-Law Dependence of Bacterial Invasion on Mammalian Host Receptors
Source: PLoS Comput Biol. 2015 Apr 16;11(4):e1004203. doi: 10.1371/journal.pcbi.1004203 (PMC4399907; doi:10.1371/journal.pcbi.1004203)
Supplement: S1 Table — (DOCX) [file pcbi.1004203.s011.docx]

- **Table S1**: Mechanisms implicated in Invasin-mediated uptake

| - **Factor** | - **Mechanism** | - **Source** |
| --- | --- | --- |
| - **Invasin** | - INV affinity | - [1–3] |
|  | - INV self-association domain | - [4] |
|  | - INV density | - [5,6] |
|  | - Competition for β_1_-integrins | - [1] |
|  | - Expression level | - [7] |
| - **β_1_-integrins** | - Density on host surface | - [1] |
|  | - Cytoplasmic tail domains | - [8,9] |
|  |  |  |
| - **Host signaling** | - Microfilament formation | - [10] |
|  | - Tyrosine kinase signaling | - [11] |
|  | - PI3-kinase | - [12] |
|  | - Focal adhesion kinase and Src kinase | - [13] |
|  | - Rac1 | - [14] |
|  | - Cas/crk | - [15] |
|  | - Protein kinase C | - [16] |
|  | - Pyk2 | - [17] |

**REFERENCES**

1. Tran Van Nhieu G, Isberg RR. Bacterial internalization mediated by beta 1 chain integrins is determined by ligand affinity and receptor density. EMBO J. 1993;12: 1887–1895.

2. Van Nhieu GT, Isberg RR. The Yersinia pseudotuberculosis invasin protein and human fibronectin bind to mutually exclusive sites on the alpha 5 beta 1 integrin receptor. J Biol Chem. 1991;266: 24367–24375.

3. Hamburger ZA, Brown MS, Isberg RR, Bjorkman PJ. Crystal Structure of Invasin: A Bacterial Integrin-Binding Protein. Science. 1999;286: 291–295. doi:10.1126/science.286.5438.291

4. Dersch P, Isberg RR. A region of the Yersinia pseudotuberculosis invasin protein enhances integrin-mediated uptake into mammalian cells and promotes self-association. EMBO J. 1999;18: 1199–1213. doi:10.1093/emboj/18.5.1199

5. Isberg RR. Discrimination between intracellular uptake and surface adhesion of bacterial pathogens. Science. 1991;252: 934–938.

6. Rankin S, Isberg RR, Leong JM. The integrin-binding domain of invasin is sufficient to allow bacterial entry into mammalian cells. Infect Immun. 1992;60: 3909–3912.

7. Nagel G, Lahrz A, Dersch P. Environmental control of invasin expression in Yersinia pseudotuberculosis is mediated by regulation of RovA, a transcriptional activator of the SlyA/Hor family. Mol Microbiol. 2001;41: 1249–1269. doi:10.1046/j.1365-2958.2001.02522.x

8. Gustavsson A, Armulik A, Brakebusch C, Fässler R, Johansson S, Fällman M. Role of the β1-integrin cytoplasmic tail in mediating invasin-promoted internalization of Yersinia. J Cell Sci. 2002;115: 2669–2678.

9. Nhieu GTV, Krukonis ES, Reszka AA, Horwitz AF, Isberg RR. Mutations in the Cytoplasmic Domain of the Integrin Chain Indicate a Role for Endocytosis Factors in Bacterial Internalization. J Biol Chem. 1996;271: 7665–7672. doi:10.1074/jbc.271.13.7665

10. Finlay BB, Falkow S. Comparison of the invasion strategies used by Salmonella cholerae-suis, Shigella flexneri and Yersinia enterocolitica to enter cultured animal cells: endosome acidification is not required for bacterial invasion or intracellular replication. Biochimie. 1988;70: 1089–1099. doi:10.1016/0300-9084(88)90271-4

11. Rosenshine I, Duronio V, Finlay BB. Tyrosine protein kinase inhibitors block invasin-promoted bacterial uptake by epithelial cells. Infect Immun. 1992;60: 2211–2217.

12. Mecsas J, Raupach B, Falkow S. The Yersinia Yops inhibit invasion of Listeria, Shigella and Edwardsiella but not Salmonella into epithelial cells. Mol Microbiol. 1998;28: 1269–1281. doi:10.1046/j.1365-2958.1998.00891.x

13. Alrutz MA, Isberg RR. Involvement of focal adhesion kinase in invasin-mediated uptake. Proc Natl Acad Sci. 1998;95: 13658–13663. doi:10.1073/pnas.95.23.13658

14. Wong K-W, Mohammadi S, Isberg RR. The Polybasic Region of Rac1 Modulates Bacterial Uptake Independently of Self-association and Membrane Targeting. J Biol Chem. 2008;283: 35954–35965. doi:10.1074/jbc.M804717200

15. Weidow CL, Black DS, Bliska JB, Bouton AH. CAS/Crk signalling mediates uptake of Yersinia into human epithelial cells. Cell Microbiol. 2000;2: 549–560. doi:10.1046/j.1462-5822.2000.00079.x

16. Uliczka F, Kornprobst T, Eitel J, Schneider D, Dersch P. Cell invasion of Yersinia pseudotuberculosis by invasin and YadA requires protein kinase C, phospholipase C-γ1 and Akt kinase. Cell Microbiol. 2009;11: 1782–1801. doi:10.1111/j.1462-5822.2009.01371.x

17. Owen KA, Thomas KS, Bouton AH. The differential expression of Yersinia pseudotuberculosis adhesins determines the requirement for FAK and/or Pyk2 during bacterial phagocytosis by macrophages. Cell Microbiol. 2007;9: 596–609. doi:10.1111/j.1462-5822.2006.00811.x
