# Supplementary material for: A Power-Law Dependence of Bacterial Invasion on Mammalian Host Receptors
Source: PLoS Comput Biol. 2015 Apr 16;11(4):e1004203. doi: 10.1371/journal.pcbi.1004203 (PMC4399907; doi:10.1371/journal.pcbi.1004203)
Supplement: S2 Table — (DOCX) [file pcbi.1004203.s012.docx]

# Table S2: Reaction terms

| - **Term** | - **Description and Notes** |
| --- | --- |
| - $k_{fA}[A][R]$ | - Antibody binding |
| - $k_{rA}[AR]$ | - Antibody unbinding |
| - $k_{fB1}[B][R]$ | - Initial binding of bacteria |
| - $k_{rB1}[B_{1}]$ | - Dissociation of singly bound bacteria |
| - $k_{fB2}[B_{1}][R]$ | - Singly bound bacteria binding to integrins |
| - $k_{rB2}[B_{m}]$ | - Dissociation of minimally bound bacteria |
| - $k_{fB3}[B_{m}][R]$ | - Minimally bound bacteria binding integrins |
| - $k_{rB3}[B_{n}]$ | - Dissociation of maximally bound bacteria |

- Variables
- R - β_1_-integrin receptors
- B - Bacteria in free, unbound state
- B_1_ - Bacteria in singly bound state
- B_m_ - Bacteria in minimally bound state
- B_n_ - Bacteria in maximally bound state
- A - β_1_-integrin antibody
- AR - Antibody-β_1_-integrin complex
