# Supplementary material for: A Power-Law Dependence of Bacterial Invasion on Mammalian Host Receptors
Source: PLoS Comput Biol. 2015 Apr 16;11(4):e1004203. doi: 10.1371/journal.pcbi.1004203 (PMC4399907; doi:10.1371/journal.pcbi.1004203)
Supplement: S3 Table — (DOCX) [file pcbi.1004203.s013.docx]

# Table S3: Ordinary Differential Equations

| - **ODEs shared by full and 3-stage model** |
| --- |
| - **$\frac{d[A]}{dt}=-k_{fA}[A][R]+k_{rA}[AR]$** |
| - **$\frac{d[AR]}{dt}=k_{fA}[A][R]-k_{rA}[AR]$** |
| - $\frac{d[B]}{dt}=-k_{fB1}[B][R]+k_{rB1}[B_{1}]$ |
| - $\frac{d[B_{1}]}{dt}=k_{fB1}[B][R]-k_{rB1}[B_{1}]-k_{fB2}[B_{1}][R]+k_{rB2}[B_{m}]$ |
| - **3-Stage model ODEs** |
| - $\begin{matrix} \frac{d[R]}{dt}=-k_{fB1}[B][R]+k_{rB1}[B_{1}]+(m-1)k_{rB2}[B_{m}]+(n-m)k_{rB3}[B_{n}] \\ -(m-1)k_{fB2}[B_{1}][R]-(n-m)k_{fB3}[B_{m}][R]+k_{rA}[AR]-k_{fA}[A][R] \end{matrix}$ |
| - $\frac{d[B_{m}]}{dt}=k_{fB2}[B_{1}][R]-k_{rB2}[B_{m}]-k_{fB3}[B_{m}][R]+k_{rB3}[B_{n}]$ |
| - $\frac{d[B_{n}]}{dt}=k_{fB3}[B_{m}][R]-k_{rB3}[B_{n}]$ |
| - **Full model ODEs** |
| - $\frac{d[R]}{dt}=-k_{fB1}[B][R]+k_{rB1}[B_{1}]+k_{rA}[AR]-k_{fA}[A][R]+\sum_{i=2}^{100} (-k_{fBi}[B_{i-1}][R]+k_{rBi}[B_{i}])$ |
| - $\begin{matrix} \frac{d[B_{i}]}{dt}=k_{fBi}[B_{i-1}][R]-k_{rBi}[B_{i}]-k_{fBi+1}[B_{i}][R]+k_{rBi+1}[B_{i+1}] \\ i=2,...,n-1 \end{matrix}$ |
| - $\frac{d[B_{n}]}{dt}=k_{fBn}[B_{n-1}][R]-k_{rBn}[B_{n}]$ |
