# Supplementary material for: A Power-Law Dependence of Bacterial Invasion on Mammalian Host Receptors
Source: PLoS Comput Biol. 2015 Apr 16;11(4):e1004203. doi: 10.1371/journal.pcbi.1004203 (PMC4399907; doi:10.1371/journal.pcbi.1004203)
Supplement: S4 Table — (DOCX) [file pcbi.1004203.s014.docx]

# Table S4: Parameters

| - Constant | - Value | - Description and source |
| --- | --- | --- |
| - *k_fB1_* | - 4.62·10^-2^ nM^-1^ min^-1^ | - On-rate for INV-integrin binding |
| - *k_rB1_* | - 3.47·10^-2^ min^-1^ | - Off-rate for INV-integrin (t_1/2_~20 min [1]) |
| - *k_fB2_* (^a^*k_fBi_*) | - 4.62·10^-2^ nM^-1^ min^-1^ | - No difference from *k_fB1_* |
| - *k_rB2_* (^a^*k_rBi_*) | - *k_rB1_*·10^-1^ min^-1^ | - Assumed to be 10 times smaller than *k_rB1_* |
| - *k_fB3_* | - 4.62·10^-2^ nM^-1^ min^-1^ | - No difference from *k_fB2_* |
| - *k_rB3_* | - *k_rB2_*·10^-2^ min^-1^ | - Assumed to be 100 times smaller than *k_rB2_* |
| - *k_fa_* | - 10^-4^ nM^-1^ min^-1^ | - Typical on rate for antibody-protein interaction |
| - *k_ra_* | - 10^-3^ min^-1^ | - Typical off-rate for antibody-protein with K_D_ = 10 nM [2] |
| - *^b^n* | - 100 | - Number of integrins bound by bacteria in maximally bound state (B_n_) |
| - *^b^m* | - 10 | - Number of integrins bound by bacteria in minimally bound state (B_m_) |

- ^a^In full model, k_fBi_ is 10-fold higher than k_fB1_ and k_rBi_ is 10-fold lower than k_rB1_ for all *i* between 2 and 100
- ^b^Chosen arbitrarily with m=n/10
- References

1. Van Nhieu GT, Isberg RR. The Yersinia pseudotuberculosis invasin protein and human fibronectin bind to mutually exclusive sites on the alpha 5 beta 1 integrin receptor. J Biol Chem. 1991;266: 24367–24375.

2. Larvor M-P, Djavadi-Ohaniance L, Nall B, Goldberg ME. Measurement of the dissociation rate constant of antigen/antibody complexes in solution by enzyme-linked immunosorbent assay. J Immunol Methods. 1994;170: 167–175. doi:10.1016/0022-1759(94)90392-1
